# Metal–Organic Frameworks in Food Biotechnology: Opportunities, Challenges, and Future Perspectives for Probiotic Delivery, Precision Fermentation, and Circular Food Systems

**DOI:** 10.3390/nano16150946

**Published:** 2026-07-31

**Authors:** Huy Loc Nguyen

**Affiliations:** Department of Engineering and Technology, Van Hien University, Ho Chi Minh City 72419, Vietnam; locnh007426@online.vhu.edu.vn

**Keywords:** metal-organic frameworks, controlled release, food systems, functional foods, microbial immobilization, precision fermentation, probiotics

## Abstract

Metal–organic frameworks (MOFs) have emerged as a versatile class of porous nanomaterials with exceptional surface area, tunable pore architectures, and customizable chemical functionalities, creating new opportunities for advanced food applications. Increasing demand for functional foods, precision fermentation, and sustainable bioprocessing has stimulated interest in MOFs as multifunctional platforms for microbial encapsulation, biocatalyst stabilization, and resource recovery. This review examines recent advances in the design and application of MOFs for probiotic delivery, precision fermentation, and circular food systems. The relationships between MOF structure, physicochemical properties, and functional performance are discussed in the context of probiotic encapsulation, protection against environmental and gastrointestinal stress, and controlled release within the intestinal tract. Emerging applications in precision fermentation are evaluated, including microbial immobilization, enzyme stabilization, metabolite separation, and bioprocess intensification. The potential of MOFs to enable circular food systems through the valorization of fermentation by-products, nutrient recovery, and waste-to-value strategies is also assessed. Despite significant progress, challenges related to biocompatibility, food-grade synthesis, scalability, regulatory approval, and long-term safety continue to limit industrial implementation. Future research directions include the development of sustainable and biodegradable MOFs, data-driven material design, and standardized evaluation frameworks to accelerate the translation of MOF-enabled technologies from laboratory research to commercial food applications.

## 1. Introduction

Probiotics are commonly defined as live microorganisms that, when administered in adequate amounts, confer health benefits to the host [1]. They have been widely incorporated into fermented dairy products, beverages, dietary supplements, and emerging functional foods because of their potential roles in modulating the gut microbiota, supporting intestinal barrier function, regulating immune responses, and producing bioactive metabolites [2]. However, the practical application of probiotics remains constrained by their susceptibility to processing and storage conditions, including oxygen exposure, temperature fluctuations, and dehydration, which can substantially reduce cell viability [3]. Moreover, probiotics encounter multiple physiological barriers following ingestion, such as acidic gastric conditions, bile salts, digestive enzymes, and competition with resident gut microorganisms, all of which can impair survival and colonization efficiency [4,5]. Consequently, maintaining adequate probiotic viability throughout manufacturing, storage, and gastrointestinal transit remains a major challenge for probiotic-based food systems [6].

Encapsulation has therefore become a central strategy for improving probiotic stability and targeted delivery [7]. Conventional encapsulation systems based on alginate, chitosan, gelatin, starch, pectin, whey proteins, and other biopolymers have shown promise in protecting probiotic cells against environmental and gastrointestinal stresses [8]. These systems can improve survival during drying, storage, fermentation, and gastric transit while enabling controlled release in the intestinal environment. Nevertheless, many conventional matrices have limitations related to weak mechanical strength, uncontrolled porosity, inconsistent release behavior, low resistance under acidic conditions, and limited capacity for multifunctional design [9]. In addition, the growing complexity of functional food development requires delivery platforms that not only protect microbial cells but also interact with food matrices, respond to local physicochemical conditions, and support broader bioprocessing goals [10].

Metal–organic frameworks (MOFs) have emerged as a highly tunable class of crystalline porous materials constructed from metal ions or clusters coordinated with organic linkers [11]. Their high surface area, adjustable pore size, structural diversity, and chemical functionality have led to extensive investigation in gas storage, separation, catalysis, sensing, drug delivery, and biomedicine. Among them, zeolitic imidazolate frameworks such as ZIF-8 have received considerable attention because of their relatively mild synthesis conditions, high porosity, and pH-responsive degradation behavior [12]. These features are attractive for biological and food-related applications because MOFs can be engineered to encapsulate or interface with biomolecules, enzymes, nutrients, antimicrobials, and living cells. Compared with many conventional encapsulation materials, MOFs offer a distinctive combination of molecular-level design, protective architecture, and stimulus-responsive behavior.

A key development in this field is biomimetic mineralization, through which metal–organic framework (MOF) shells can be formed around biological entities under mild conditions [13]. Early studies demonstrated that MOFs could serve as protective coatings for biomacromolecules, enhancing their stability while preserving biological activity [14]. Subsequent investigations extended this concept to living cells, showing that ZIF-8 coatings function as cytoprotective exoskeletons that physically shield microorganisms while allowing the diffusion of nutrients and metabolites necessary for cell survival [15]. This concept is highly relevant to probiotic delivery because MOF-based coatings may protect cells during processing, storage, and gastrointestinal transit while enabling triggered release under specific environmental conditions [16]. More recently, researchers have explored biocompatible MOF matrices beyond ZIF-8, including iron fumarate-based systems such as MIL-88A, for encapsulating probiotic strains including *Lactiplantibacillus plantarum* [17].

The application of MOFs to probiotic systems is still at an early stage compared with their use in drug delivery and enzyme immobilization. Most probiotic delivery research has traditionally focused on polymeric microcapsules, hydrogels, emulsions, and biopolymer-based coatings, including alginate, chitosan, proteins, and polysaccharide systems [18]. MOF-based probiotic encapsulation introduces new possibilities but also raises important questions. The chemical composition of the metal nodes and organic linkers must be carefully selected to ensure biocompatibility, biodegradability, and relevance for oral or food-related applications [19]. The synthesis conditions must preserve cell viability, and the resulting coatings must balance protection with nutrient diffusion and controlled release. In addition, MOF degradation products, potential metal ion release, long-term safety, and regulatory acceptability must be evaluated before food applications can be realized. These issues are especially important because food systems involve repeated exposure, diverse consumer populations, and complex matrices that differ from many biomedical delivery contexts [20].

Beyond probiotic delivery, MOFs may also contribute to precision fermentation. Precision fermentation uses microorganisms as programmable cell factories to produce targeted food ingredients, including proteins, enzymes, flavor compounds, lipids, vitamins, pigments, and other high-value biomolecules [21]. This approach is gaining attention as a sustainable alternative to conventional animal-derived ingredients and as a tool for producing functional compounds with improved consistency and resource efficiency. However, precision fermentation still faces challenges related to cell stress, biocatalyst instability, product inhibition, downstream separation, productivity, and process cost [22]. Materials that can stabilize cells or enzymes, improve mass transfer, selectively adsorb metabolites, or facilitate product recovery may help improve fermentation efficiency and scalability [23].

MOFs are particularly relevant to this area because they have been extensively investigated as supports for enzyme immobilization and biocatalysis [24]. Enzyme@MOF systems can enhance enzyme stability, reusability, resistance to harsh reaction conditions, and catalytic efficiency by confining enzymes within porous or protective frameworks [25]. In fermentation-related applications, similar design principles could be applied to immobilize enzymes involved in substrate conversion, stabilize sensitive biocatalysts, protect microbial cells, or remove inhibitory compounds from fermentation media [26]. MOF-based materials may also serve as selective adsorbents for metabolite capture and purification, thereby reducing downstream processing burdens [27]. These functions align well with the goals of precision fermentation, where improved control over microbial performance and product recovery is essential.

The relationship between MOFs and circular food systems represents another emerging opportunity. Food and fermentation industries generate large quantities of residues, including spent biomass, protein-rich side streams, carbohydrate-rich wastes, organic acids, minerals, and process effluents [28]. Circular food systems aim to reduce waste, recover nutrients, and convert low-value residues into functional ingredients, energy, fertilizers, or platform chemicals. Fermentation is already a key technology for valorizing food residues, but process efficiency is often limited by variable feedstock composition, inhibitory compounds, low product recovery, and the need for selective separation [29,30]. MOFs may support circularity by enabling nutrient recovery, adsorption of valuable metabolites, catalytic conversion of waste-derived compounds, immobilization of microorganisms for repeated fermentation cycles, and stabilization of enzymes used in waste-to-value processes [31].

Despite these opportunities, the integration of MOFs into food, probiotic, and fermentation systems requires a careful evaluation of material safety, sustainability, and translational feasibility. Many MOFs are synthesized using organic solvents, non-food-grade linkers, or metal ions that may not be acceptable for food use [32]. The development of water-based, low-energy, biocompatible, biodegradable, and food-grade MOF synthesis routes is therefore essential. The selection of safer metal nodes, such as iron, calcium, magnesium, zinc, or other nutritionally relevant elements, may improve translational potential, but dose, bioavailability, toxicity, and release kinetics must still be assessed [33,34]. Similarly, the environmental footprint of MOF synthesis, regeneration, and disposal must be considered if these materials are proposed for circular food systems.

Current literature on MOFs in food-related contexts is distributed across several separate areas, including oral delivery of bioactive compounds, enzyme immobilization, microbial encapsulation, antimicrobial materials, food packaging, sensing, and waste valorization [35]. However, limited attention has been given to connecting MOF-enabled probiotic delivery with precision fermentation and circular food system design. This connection is important because these areas share common material requirements: biocompatibility, controlled transport, protection of biological function, responsiveness to environmental conditions, and compatibility with scalable processing [36,37]. A review that integrates these themes can help identify how MOFs may move beyond isolated laboratory demonstrations toward multifunctional platforms for food biotechnology.

Therefore, this review examines recent advances and future opportunities in metal–organic frameworks for probiotic delivery, precision fermentation, and circular food systems. The discussion focuses on MOF design principles, probiotic encapsulation mechanisms, gastrointestinal protection and release, microbial and enzyme immobilization, fermentation process intensification, waste valorization, safety considerations, and translational barriers. By linking material structure with biological function and food-system sustainability, this review aims to clarify the current state of the field and highlight research directions needed to develop MOF-enabled technologies for next-generation functional foods and sustainable bioprocessing.

## 2. Methodologies

This review was designed as a narrative and critical review to synthesize current knowledge on the application of metal–organic frameworks (MOFs) in probiotic delivery, precision fermentation, and circular food systems. Because the topic integrates materials science, food biotechnology, microbiology, and sustainable bioprocessing, a broad literature search strategy was adopted to capture both fundamental MOF studies and recent food-related applications. The review focused on identifying how MOF structure, composition, porosity, surface chemistry, biocompatibility, and stimulus-responsive behavior influence their potential use in probiotic protection, microbial immobilization, enzyme stabilization, fermentation process enhancement, and waste valorization.

### 2.1. Literature Search Strategy

Relevant publications were collected from major scientific databases, including Web of Science, Scopus, PubMed, ScienceDirect, SpringerLink, ACS Publications, Wiley Online Library, MDPI, and Google Scholar. The literature search primarily covered articles published from 2014 to 2026, while earlier foundational papers on MOF chemistry, probiotic definitions, and biomimetic mineralization were also included when they provided essential background. The search was conducted using combinations of keywords related to three main themes: MOF-based materials, probiotic and microbial delivery systems, and fermentation or circular food applications.

The following search terms were used individually and in combination: “metal organic frameworks”, “MOFs”, “ZIF-8”, “MIL-88A”, “biocompatible MOFs”, “food-grade MOFs”, “MOF encapsulation”, “biomimetic mineralization”, “probiotic encapsulation”, “probiotic delivery”, “living cell encapsulation”, “microbial immobilization”, “enzyme immobilization”, “precision fermentation”, “fermentation technology”, “functional foods”, “food biotechnology”, “fermentation waste valorization”, “circular food systems”, “nutrient recovery”, and “sustainable bioprocessing”. Boolean operators such as AND and OR were used to refine the search. For example, search strings included “MOFs AND probiotics”, “ZIF-8 AND probiotic encapsulation”, “metal organic frameworks AND microbial immobilization”, “MOFs AND precision fermentation”, and “MOFs AND food waste valorization”.

### 2.2. Inclusion and Exclusion Criteria

Studies were selected based on their relevance to MOF design, biological encapsulation, food-related functionality, fermentation applications, or circular bioeconomy strategies. Research articles, review articles, book chapters, and authoritative reports were considered when they provided useful information on MOF synthesis, probiotic delivery, enzyme immobilization, microbial protection, fermentation intensification, metabolite recovery, or food waste valorization.

Articles were included if they met at least one of the following criteria:

(i) They reported the synthesis, characterization, or functional modification of MOFs relevant to biological or food applications;
(ii) They investigated MOFs for probiotic, microbial, enzyme, or biomolecule encapsulation;
(iii) They discussed biocompatible, biodegradable, or food-relevant MOF systems;
(iv) They examined MOFs in controlled release, gastrointestinal delivery, or biological protection;
(v) They evaluated MOFs for microbial immobilization, biocatalysis, or fermentation-related processes;
(vi) They addressed waste valorization, nutrient recovery, or circular food-system applications involving porous materials or MOF-based platforms.

Studies were excluded if they focused exclusively on non-food applications without transferable relevance to probiotics, fermentation, or sustainable food systems. Articles dealing only with gas storage, energy storage, electronic devices, or purely inorganic catalysis were not prioritized unless their material design principles were directly applicable to biological or food-related MOF systems. Studies lacking sufficient methodological detail, material characterization, or relevance to the scope of this review were also excluded.

### 2.3. Screening and Selection Process

The collected literature was first screened by title and abstract to determine relevance to the review scope. Articles that appeared relevant were then examined in full to assess their scientific contribution, experimental design, and connection to the major themes of the review. Special attention was given to studies that reported clear relationships between MOF composition, structure, physicochemical properties, and biological performance. For probiotic and microbial applications, key factors included cell viability, encapsulation efficiency, stress protection, gastrointestinal stability, release behavior, and potential cytotoxicity. For fermentation-related applications, attention was given to microbial immobilization, enzyme stability, catalytic performance, reusability, metabolite adsorption, product recovery, and process scalability.

Because the field of MOF-enabled probiotic and fermentation technologies is still emerging, the review also included relevant studies from adjacent areas, such as enzyme@MOF biocatalysis, biomolecule encapsulation, oral drug delivery, antimicrobial food materials, and food waste valorization. These studies were used to identify transferable mechanisms and design principles that may guide future development of MOF-based platforms for food biotechnology.

### 2.4. Data Extraction and Thematic Organization

Information was extracted from selected publications and organized according to material type, biological target, application area, and reported performance. For MOF-based materials, extracted information included metal nodes, organic linkers, framework type, synthesis method, particle size, porosity, surface charge, morphology, stability, degradation behavior, and functional modification. For probiotic and microbial studies, extracted data included microbial species, encapsulation approach, viability outcomes, stress tolerance, gastrointestinal protection, and release profile. For enzyme and fermentation studies, extracted information included enzyme or microbial system, immobilization strategy, catalytic activity, operational stability, reusability, product yield, and separation performance.

The selected literature was then grouped into major thematic areas:

(i) Fundamental properties of MOFs relevant to food biotechnology;
(ii) MOF-based probiotic encapsulation and controlled delivery;
(iii) MOF-enabled microbial and enzyme stabilization for precision fermentation;
(iv) MOF applications in metabolite separation, nutrient recovery, and fermentation waste valorization;
(v) Safety, regulatory, and scalability considerations.

This structure was used to integrate evidence from different disciplines and to identify research gaps that limit translation from laboratory studies to practical food applications.

### 2.5. Critical Analysis Approaches

Rather than simply summarizing individual studies, this review applied a critical synthesis approach to evaluate how MOF properties influence biological and technological performance. The discussion considered both advantages and limitations of MOF-based systems compared with conventional encapsulation and immobilization materials. Key evaluation criteria included biocompatibility, food-grade potential, synthesis conditions, protection efficiency, controlled release behavior, interaction with food matrices, stability during processing and storage, scalability, cost, environmental impact, and regulatory feasibility.

Particular attention was given to the distinction between proof-of-concept demonstrations and technologies with realistic potential for food applications. For example, MOFs synthesized using harsh solvents, toxic metal ions, or non-food-compatible linkers were evaluated cautiously, even when they showed promising technical performance. In contrast, water-based synthesis, mild reaction conditions, nutritionally relevant metal nodes, biodegradable linkers, and demonstrated biological safety were considered important indicators of translational potential.

### 2.6. Limitations of the Review Methodology

This review has several limitations. First, the application of MOFs in probiotic delivery and precision fermentation remains relatively new; therefore, the number of studies directly addressing food-grade MOF systems for live probiotic delivery is still limited. As a result, some conclusions were drawn from related fields, including enzyme immobilization, biomimetic mineralization, and oral biomolecule delivery. Second, differences in experimental design, microbial strains, MOF composition, exposure conditions, and viability assessment methods make direct comparison among studies difficult. Third, many studies remain at the laboratory scale and do not provide sufficient information on long-term safety, sensory impact, regulatory approval, industrial scalability, or environmental fate. These limitations highlight the need for standardized evaluation protocols and more application-specific studies before MOF-based systems can be fully translated into food and fermentation technologies.

### 2.7. Scope of the Review

This review focuses on MOFs and MOF-based nanoarchitectures with potential relevance to probiotic delivery, precision fermentation, and circular food systems. It does not aim to provide a complete review of all MOF applications in food science, such as food packaging, contaminant sensing, or antimicrobial coatings, although selected examples from these areas are discussed when they provide useful design principles. The main objective is to connect material design with biological function and sustainable food-system applications, thereby identifying how MOFs may contribute to next-generation functional foods, controlled microbial delivery, advanced fermentation processes, and waste-to-value strategies.

## 3. Design Principles and Functional Properties of Metal–Organic Frameworks for Food Biotechnology

### 3.1. Structural Characteristics of MOFs Relevant to Food and Biological Systems

Metal–organic frameworks (MOFs) are crystalline porous materials constructed through coordination interactions between metal ions or metal clusters and organic linkers. Their development has significantly expanded the design space of porous materials because both the inorganic and organic components can be selected, modified, and spatially organized with high structural precision [38]. This modularity distinguishes MOFs from conventional porous materials such as zeolites, activated carbon, mesoporous silica, and polymeric matrices, which generally offer less flexibility in tuning pore chemistry, framework composition, and interfacial functionality [39]. For food biotechnology, these features are especially valuable because materials must operate within chemically complex environments containing water, salts, proteins, polysaccharides, organic acids, phenolics, lipids, metabolites, enzymes, and living microorganisms.

**Figure 1:**
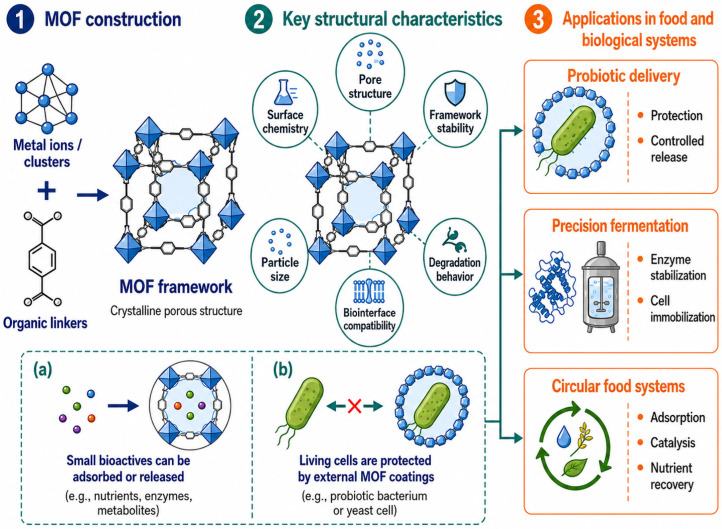
Key Structural Attributes of Metal–Organic Frameworks in Food Biotechnology.

The suitability of MOFs for probiotic delivery, precision fermentation, and circular food systems is largely governed by their pore structure, surface chemistry, framework stability, particle size, degradation behavior, and compatibility with biological interfaces [40]. High internal surface area and adjustable porosity allow MOFs to adsorb, stabilize, or release small bioactive molecules, nutrients, enzymes, antimicrobial agents, and fermentation-derived metabolites [41]. However, when the target is a living probiotic cell, the role of MOFs differs fundamentally from conventional cargo loading. Bacterial and yeast cells are much larger than MOF micropores; therefore, MOFs generally function as external protective shells, interfacial coatings, or microenvironment-modulating matrices rather than as internal containers for whole cells [42]. This distinction is important because successful probiotic delivery requires preservation of cell viability, nutrient exchange, metabolic function, and release behavior, rather than maximization of loading capacity alone. The key structural features that determine the performance of MOFs in food and biological systems are summarized in Figure 1, highlighting the distinction between small-molecule adsorption within MOF pores and external MOF-based protection of living microbial cells.

Among MOF families, zeolitic imidazolate frameworks, particularly ZIF-8, have been extensively investigated as model systems for biological encapsulation because they can be synthesized under relatively mild conditions and exhibit pH-responsive degradation [43]. ZIF-8, composed of zinc ions and 2-methylimidazole, possesses high porosity, good stability under near-neutral conditions, and acid-sensitive disassembly, which has encouraged its use in oral delivery and biomimetic coating studies [44]. These characteristics are attractive for food and gastrointestinal applications in which a material may need to remain stable during processing or storage but disassemble under specific biological conditions. Nevertheless, the use of ZIF-8 in food applications requires careful evaluation because zinc release, imidazolate exposure, particle persistence, and repeated dietary intake may influence biocompatibility and regulatory acceptance [45].

A broader range of MOF chemistries is therefore needed for food-oriented applications. Iron-based MOFs, including MIL-88A, MIL-100(Fe), and MIL-101(Fe), have attracted interest because iron is biologically relevant and can be coordinated with carboxylate linkers [46]. MIL-88A, formed from iron and fumaric acid, is particularly relevant because fumaric acid is a food-related organic acid and recent work has explored this framework for encapsulating *Lactiplantibacillus plantarum* [47]. Zirconium-based MOFs such as UiO-66 offer exceptional chemical stability, but their translation into food systems depends on the safety of the linker, degradation products, and potential long-term exposure [48,49]. Calcium-, magnesium-, zinc-, and iron-based MOFs may provide stronger food relevance than frameworks based on metals with limited dietary acceptability, although safety must still be assessed in relation to dose, chemical speciation, release kinetics, and matrix interactions [45,50].

For the scope of this review, metal–organic frameworks (MOFs) should be understood not only as porous carriers but also as programmable interfaces between materials and biological function. In probiotic delivery, MOFs may protect microorganisms against acid, oxygen, dehydration, heat, bile salts, and antimicrobial compounds [18]. In precision fermentation, they may stabilize enzymes, immobilize cells, enhance substrate conversion, adsorb inhibitory compounds, or facilitate downstream product recovery [51]. In circular food systems, their adsorption, catalytic, and separation properties may contribute to nutrient recovery, waste valorization, and reuse of fermentation-derived streams [52]. These diverse applications are connected by a common materials design principle: biological outcomes depend on how framework composition, surface chemistry, transport behavior, and degradation are coordinated within specific food or bioprocessing environments.

### 3.2. Synthesis Strategies for Biocompatible and Food-Relevant MOFs

The synthesis method strongly determines the biological suitability of MOFs because it influences crystallinity, particle size, morphology, defect density, residual solvent content, surface chemistry, and framework stability [53]. Traditional solvothermal synthesis has been essential for developing highly crystalline MOFs, but the use of organic solvents, elevated temperature, prolonged reaction time, and intensive purification can limit direct application in food biotechnology [54]. Probiotic delivery and fermentation-related applications require synthesis routes that are compatible with aqueous media, mild pH, moderate temperature, and biological activity. Therefore, food-relevant MOF development must move beyond structural novelty and address whether the synthesis process itself is safe, scalable, reproducible, and compatible with living systems.

Aqueous and room-temperature synthesis routes are particularly important for this purpose. ZIF-8 and selected iron-carboxylate frameworks can be prepared under mild conditions, making them useful candidates for biological encapsulation and food-related studies [55]. Water-based synthesis reduces solvent residues and improves compatibility with probiotics, enzymes, proteins, and polysaccharide matrices. At the same time, aqueous synthesis may modify nucleation and growth kinetics, resulting in changes in particle size, crystallinity, shell density, and degradation behavior [56]. These changes are not merely synthetic details; they directly affect microbial viability, molecular diffusion, acid resistance, and release performance. In probiotic encapsulation, for example, a thin and porous MOF layer may allow nutrient and metabolite exchange while providing stress protection, whereas a dense or excessively thick shell may inhibit cell metabolism or delay release after ingestion [57,58].

Biomimetic mineralization has become one of the most influential approaches for integrating MOFs with biological materials. In this strategy, biomolecules or cells serve as nucleation templates for MOF formation under mild conditions. Liang et al. (2015) [12] demonstrated that biomimetic MOF mineralization can generate protective coatings around biomacromolecules and improve their stability. The same concept was later extended to living cells, where MOF shells acted as cytoprotective exoskeletons. This approach is highly relevant to probiotics because bacterial surfaces naturally contain functional groups capable of interacting with metal ions and linkers, including carboxyl, phosphate, hydroxyl, and amine groups associated with proteins, polysaccharides, teichoic acids, and lipopolysaccharides [59]. These interactions can promote local nucleation and framework growth around the cell envelope.

One-pot encapsulation provides a comparatively simple route for forming MOF–bio composites by combining cells or biomolecules with metal ions and organic linkers in a single synthesis step. Its operational simplicity is attractive for scale-up, but reaction conditions must be carefully optimized to avoid compromising the integrity and functionality of the encapsulated biological entity [51]. Metal ion concentration, ligand concentration, reaction time, pH, ionic strength, and post-synthesis washing conditions can all influence encapsulation efficiency, framework characteristics, and cell viability [60]. This consideration is particularly important for probiotic systems because preservation of colony-forming ability alone is insufficient; encapsulated microorganisms must also retain stress tolerance, metabolic activity, and functional performance following release. Recent work on the encapsulation of *Lactiplantibacillus plantarum* within the iron fumarate framework MIL-88A illustrates the growing interest in MOFs with more biocompatible compositions and milder synthesis conditions [18]. To improve reproducibility and facilitate future food applications, these synthesis parameters should be systematically reported and optimized, together with quantitative information on precursor concentrations, metal-to-ligand ratios, encapsulation efficiency, residual metal content, and cytocompatibility. Such standardized reporting would enable meaningful comparison among studies and support the development of reproducible and food-relevant MOF synthesis protocols.

Post-synthetic modification and composite formation can further improve the compatibility of metal–organic frameworks (MOFs) with food systems. Surface functionalization or coating with biopolymers such as chitosan, alginate, hyaluronic acid, pectin, starch, cellulose derivatives, gelatin, proteins, and polyphenols can enhance colloidal stability, reduce direct exposure to framework components, tailor surface charge, and regulate degradation and release behavior [61]. MOF–biopolymer hybrids are particularly attractive because they combine the structural tunability and stimulus responsiveness of MOFs with the biocompatibility, biodegradability, and processability of food-derived polymers [32]. In probiotic applications, these composites may improve protection during drying, storage, gastric transit, and bile exposure. In fermentation applications, polymer-modified MOFs may improve dispersion in aqueous environments, reduce aggregation, and facilitate catalyst recovery and reuse [62,63].

Although no universally accepted quantitative criteria currently exist for food-grade MOF synthesis, establishing standardized synthesis windows will be important for future translation. Rather than relying on fixed threshold values, future studies should report representative precursor molarities, metal-to-ligand ratios, reaction yields, purification procedures, and residual precursor levels using standardized protocols. These parameters should be evaluated together with cytotoxicity, microbial viability, and framework reproducibility to identify synthesis conditions that minimize biological risk while maintaining structural integrity and functional performance. Such harmonized reporting will facilitate comparison among studies and accelerate the development of safe and reproducible MOF platforms for food biotechnology.

Sustainable synthesis is also becoming increasingly important. Mechanochemical synthesis, microwave-assisted synthesis, solvent-free processing, and the use of renewable or bio-derived ligands have been proposed to reduce environmental burdens and improve scalability [56]. For circular food systems, the sustainability of MOF production must be considered alongside the sustainability of the intended application. Materials designed for waste valorization or nutrient recovery offer limited translational value if they require toxic solvents, scarce metals, energy-intensive manufacturing, or complex regeneration processes [53]. Future food-grade MOFs should therefore prioritize mild aqueous synthesis, low-toxicity precursors, minimal purification requirements, and compatibility with existing food and fermentation processing infrastructure [64].

### 3.3. Pore Architecture, Surface Chemistry, and Transport Behavior

The performance of MOFs in food biotechnology depends strongly on how their pore architecture and surface chemistry regulate molecular transport. Pore size, aperture diameter, framework flexibility, hydrophilicity, defect structure, and internal chemical environment determine whether specific molecules can enter, adsorb, diffuse, or be released [65]. In small-molecule delivery, these parameters govern the loading and release of vitamins, antioxidants, polyphenols, antimicrobial compounds, flavor molecules, and other bioactives [66]. In enzyme immobilization, pore architecture influences whether enzymes can be confined inside mesoporous cavities, adsorbed on external surfaces, or encapsulated through in situ framework growth [67]. For probiotic cells, MOF pores do not accommodate the whole organism, but they may control the diffusion of protons, bile salts, nutrients, metabolites, water, and protective compounds across the coating [68].

Surface chemistry determines how MOFs interact with microbial envelopes, proteins, polysaccharides, and food components. Surface charge influences cell adhesion, colloidal stability, aggregation, and interaction with mucus or matrix biopolymers [69]. Positively charged MOFs may nucleate efficiently on negatively charged bacterial surfaces, but excessive cationic interaction may also disrupt membranes or impair growth [70]. Polymer-coated or negatively charged systems may show improved biocompatibility and dispersion but may require additional strategies to maintain coating efficiency. Hydrophilic surfaces generally improve compatibility with aqueous food systems, whereas more hydrophobic domains may favor interaction with lipophilic molecules or protect moisture-sensitive cargo [71]. The optimal surface profile therefore depends on the intended biological function and matrix environment.

Controlled transport is particularly important for living-cell encapsulation. A MOF shell must restrict harmful stressors while allowing sufficient exchange of nutrients, metabolites, gases, and other small molecules required to maintain cell viability. If mass transfer is excessively limited, the coating may suppress cellular metabolism, delay recovery, or reduce functionality after release. Conversely, if the framework is overly permeable or unstable, protection during processing, storage, and gastrointestinal transit may be inadequate [72]. Current evidence from MOF-coated living cells indicates that thin, porous shells can provide effective physical protection while preserving the diffusion of essential small molecules [73]. However, probiotic applications require strain-specific optimization because cell wall architecture, stress sensitivity, metabolic requirements, and surface chemistry vary substantially among *Lactiplantibacillus*, *Lacticaseibacillus*, *Bifidobacterium*, *Saccharomyces*, and other microorganisms used in food fermentation and probiotic formulations [74].

Framework flexibility and pH-responsive degradation further expand the design possibilities of metal–organic frameworks (MOFs). MIL-88-type materials exhibit breathing behavior, in which pore dimensions and framework volume reversibly change in response to guest molecules or environmental conditions [75]. This flexibility may facilitate tunable uptake and release but can also complicate predictions of framework stability and transport behavior. Zeolitic imidazolate framework-8 (ZIF-8) and related systems are particularly attractive because their pH-sensitive degradation enables triggered release under specific environmental conditions [35]. In probiotic delivery, however, the desired degradation profile must be carefully tailored. Many probiotic microorganisms require protection during gastric transit and subsequent release in the intestine; therefore, materials that degrade rapidly under acidic gastric conditions may require additional protective layers or composite designs to achieve effective delivery [76]. In contrast, fermentation applications often require prolonged structural stability, repeated use, or sustained adsorption performance rather than rapid framework disassembly.

The degradation and transport behavior of MOFs should also be interpreted using appropriate kinetic terminology. Depending on the experimental system, framework disassembly, cargo release, or adsorption processes may follow different kinetic models, and the selected model should reflect the underlying physicochemical mechanism rather than merely providing the best mathematical fit. In particular, pseudo-first-order and pseudo-second-order kinetic models are empirical descriptions that should not be confused with true reaction orders derived from elementary chemical rate laws. Future studies should therefore clearly justify the choice of kinetic model and report the corresponding experimental conditions to facilitate meaningful comparisons among MOF systems and improve mechanistic understanding of their transport and degradation behavior.

Defects within MOF structures add another layer of functionality. Missing-linker and missing-cluster defects can generate open metal sites, increase mesoporosity, modify water stability, and enhance mass transport properties [77]. These characteristics may improve enzyme immobilization, metabolite adsorption, and catalytic transformation. However, defect-rich frameworks may also exhibit increased metal ion release, altered degradation behavior, and reduced structural consistency [40]. For food-related applications, defect engineering should therefore be evaluated not only in terms of performance enhancement but also with respect to reproducibility, safety, and long-term stability under relevant processing and gastrointestinal conditions.

### 3.4. Interactions Between MOFs, Biomolecules, and Living Microorganisms

**Figure 2:**
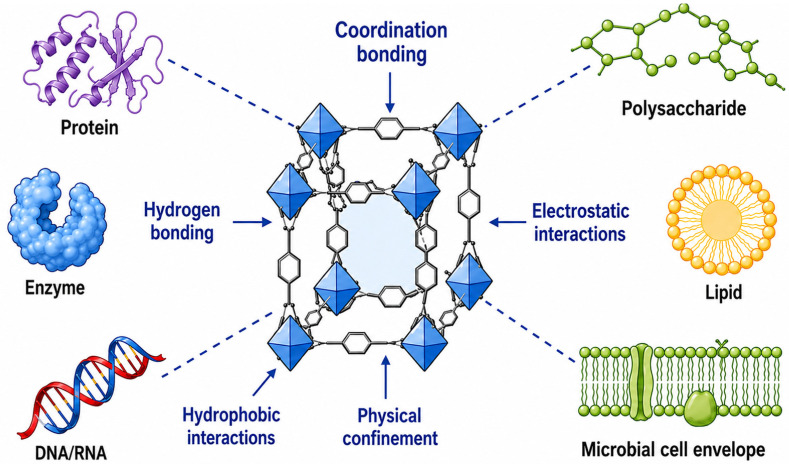
MOF–bio interaction mode.

MOF–bio interactions are central to the use of MOFs in probiotic delivery and precision fermentation. MOFs can interact with proteins, enzymes, nucleic acids, polysaccharides, lipids, and microbial cell envelopes through coordination bonding, hydrogen bonding, electrostatic interactions, hydrophobic interactions, and physical confinement [78]. These interactions may stabilize biological structures and protect them against denaturation, enzymatic degradation, acid stress, or thermal damage (Figure 2). At the same time, inappropriate interactions can reduce biological activity by altering protein conformation, damaging cell membranes, limiting substrate diffusion, or disrupting metabolic function.

Enzyme@MOF biocomposites provide a strong conceptual foundation for fermentation applications. Enzymes can be integrated with MOFs through surface adsorption, pore infiltration, covalent attachment, in situ encapsulation, or biomimetic mineralization [51]. Immobilization may enhance thermal stability, pH tolerance, storage stability, solvent resistance, and reusability. These benefits are relevant to food fermentation because enzymes are often involved in substrate hydrolysis, lactose conversion, phenolic transformation, flavor development, protein modification, and waste valorization [79]. MOF-based immobilization can also create favorable microenvironments that protect enzymes from proteolysis or harsh processing conditions. However, enzyme activity may decrease if the framework restricts substrate diffusion, causes conformational stress, or promotes leaching during repeated use [24]. Therefore, enzyme@MOF systems must be designed around the size of the enzyme, the molecular dimensions of the substrate, the polarity of the pore environment, and the intended operating conditions.

**Figure 3:**
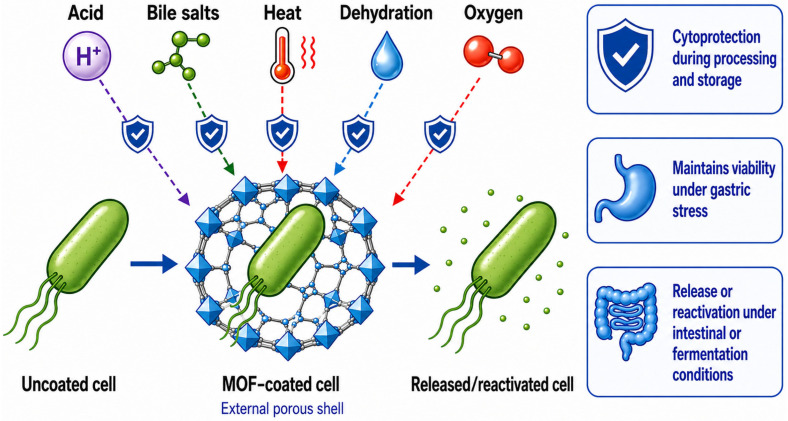
Protective mechanism of MOF coatings for enhancing probiotic survival during processing, storage, and gastrointestinal transit.

Living-cell encapsulation introduces additional complexity because cells are dynamic biological systems rather than static molecular cargo. MOF shells can improve cytoprotection, but they may also change cell-surface properties, reduce division, alter communication with the surrounding medium, or influence metabolic output [80]. For probiotics, temporary reduction in metabolic activity during storage may be beneficial if it improves stability, but the coating must permit recovery of cell function after ingestion [81]. The ideal MOF interface should protect microorganisms during processing, drying, storage, and gastric exposure, while enabling release or reactivation under intestinal or fermentation-relevant conditions (Figure 3).

The outcome of MOF coating depends strongly on microbial species and strain. Gram-positive bacteria contain thick peptidoglycan layers and teichoic acids, Gram-negative bacteria contain an outer membrane rich in lipopolysaccharides, and yeasts possess glucan- and mannan-rich cell walls [82]. These structural differences influence MOF nucleation, coating uniformity, stress tolerance, and interaction with metal ions or linkers. Although model organisms have provided useful mechanistic insight, food applications require more work with commercially and clinically relevant probiotic strains, including *Lactiplantibacillus plantarum*, *Lacticaseibacillus rhamnosus*, *Lactobacillus acidophilus*, *Bifidobacterium animalis*, *Bifidobacterium longum*, and *Saccharomyces boulardii* [83].

Food matrix interactions further complicate MOF performance. Proteins, polysaccharides, salts, organic acids, lipids, phenolics, and fermentation metabolites can adsorb onto MOF surfaces, compete for active sites, alter aggregation, or change framework stability [75]. In fermented foods, lactic acid, peptides, exopolysaccharides, and microbial metabolites may modify the behavior of MOF particles or coatings. These interactions may improve biocompatibility by forming protective coronas, but they may also block pores, slow release, or reduce adsorption capacity [84]. Consequently, MOF performance should be evaluated in realistic food and fermentation matrices rather than only in simplified buffer systems.

### 3.5. Biocompatibility and Food-Grade Design Considerations

The translation of MOFs into food biotechnology depends on rigorous assessment of biocompatibility, exposure route, and material fate. Although many MOFs show promising performance in drug delivery, catalysis, biosensing, and enzyme stabilization, food applications involve different expectations because exposure may be repeated, chronic, and population-wide. Potential concerns include metal ion release, ligand toxicity, residual solvent, nanoparticle accumulation, oxidative stress, inflammatory responses, microbiota disruption, and interactions with dietary components [85,86]. These risks do not preclude food applications, but they require a design framework that places safety and degradability alongside material performance.

Food-relevant MOFs should begin with careful selection of metal nodes and organic linkers. Nutritionally relevant metals such as iron, zinc, calcium, and magnesium may be more acceptable than metals with limited dietary safety profiles, but their biological effects depend on dose, release kinetics, chemical speciation, and matrix interactions [87]. Organic linkers also require careful selection. Fumaric acid, cyclodextrins, amino acids, peptides, and selected food-derived polyphenols may provide more favorable translational potential than synthetic linkers with limited toxicological data [88]. Even when individual components are considered safe, the assembled framework and its degradation products must be evaluated as a distinct material system.

Residual precursors and solvents are particularly important for food applications. MOF synthesis often requires washing and activation to remove unreacted ligands, guest molecules, and solvents [89]. For edible systems, purification must be efficient and analytically verified. Characterization should therefore extend beyond crystallinity, morphology, and porosity to include residual metal content, linker release, solvent residues, degradation products, and behavior under simulated digestion. In probiotic systems, cell viability should be complemented with assays for membrane integrity, metabolic activity, acid and bile tolerance, release behavior, and strain-specific functionality [90].

Particle size also influences safety and application route. Nanoscale MOFs offer high surface area and strong biological interaction, but they may raise concerns regarding intestinal uptake, translocation, persistence, or accumulation [91]. Immobilized or micron-scale MOF systems used as fermentation supports may involve lower direct consumer exposure than edible probiotic delivery systems. Therefore, safety evaluation should distinguish among edible carriers, food-contact materials, processing aids, immobilized reactor supports, and waste-treatment materials. Each application presents different exposure pathways, regulatory requirements, and acceptable risk thresholds.

### 3.6. Hybrid MOF Nanoarchitectures for Food Biotechnology

Hybrid MOF systems offer a practical route to adapt rigid porous frameworks to the soft, hydrated, and chemically diverse conditions of food biotechnology. Pure MOFs provide porosity, crystallinity, and responsive behavior, but they may lack the flexibility, processability, or biological softness required for direct interaction with probiotics and food matrices [92]. Combining MOFs with natural polymers, proteins, lipids, cellulose, hydrogels, or other biocompatible materials can improve dispersibility, mechanical stability, matrix compatibility, and controlled release [93]. Tian et al. (2024) developed moisture-responsive hydrogel beads incorporating essential oils within metal–organic frameworks (MOFs) to preserve fresh-cut pineapple [94]. The MOF-based system enabled controlled release of antimicrobial compounds under high-humidity storage conditions, improving release efficiency compared with free essential oils. Treated pineapple samples exhibited lower microbial counts, reduced weight loss, delayed softening, and improved cell membrane integrity during refrigerated storage.

Biopolymer-MOF composites are especially attractive for probiotic delivery. Chitosan can provide mucoadhesion, film-forming capacity, and antimicrobial activity, while alginate offers mild gelling and acid protection [95]. Pectin, starch, gelatin, whey proteins, and cellulose derivatives can further tune texture, digestibility, water retention, and release behavior [96]. When combined with MOFs, these polymers may reduce direct exposure of cells to metal nodes or linkers while improving protection against gastric acid, bile salts, drying, and oxidative stress [97]. However, polymer layers can also alter pore accessibility, mass transfer, and framework degradation. Thus, the design of MOF–biopolymer hybrids must balance protection, diffusion, release, and manufacturability.

MOF-hydrogel systems are another promising architecture for probiotic and fermentation applications. Hydrogels can maintain hydrated microenvironments that support cell viability, while MOFs can introduce adsorption capacity, molecular selectivity, enzyme stabilization, or stimulus responsiveness [98]. In probiotic delivery, MOF-containing hydrogels could protect cells through multiple barriers and release them in response to gastrointestinal triggers [99]. In fermentation, they could immobilize cells or enzymes while permitting transport of substrates and products. Honarmandrad et al. (2025) created a pseudo-deep eutectic solvent-coated MOF (NH_2_-UiO-66@pseudo-DES) to remove fermentation inhibitors generated during biomass hydrolysis [100]. Under optimized conditions, the material achieved removal efficiencies of 62.1% for hydroquinone, 56.1% for 5-hydroxymethylfurfural, 45.3% for furfural, and 83.5% for vanillin in synthetic hydrolysates. Similar performance was observed in real hydrolysates before fermentation. The enhanced adsorption was attributed to hydrogen bonding, electrostatic interactions, van der Waals forces, and π–π stacking. The composite maintained effective performance over four reuse cycles, demonstrating that MOF-based detoxification can improve hydrolysate quality and support more efficient microbial fermentation.

Multifunctional MOF architectures may also enable synbiotic and bioprocessing strategies. A single platform could protect probiotics while co-delivering prebiotics, antioxidants, vitamins, polyphenols, or enzymes [101]. In fermentation systems, MOF-based composites could simultaneously stabilize enzymes, mitigate microenvironmental stress, adsorb inhibitory metabolites, and facilitate product recovery [102]. However, increasing formulation complexity should be justified by demonstrable improvements in performance, as each additional component introduces challenges related to characterization, safety assessment, regulatory approval, and large-scale manufacturing.

### 3.7. Structure–Function Evaluation for Food Biotechnology Applications

For MOFs to advance from proof-of-concept materials to practical food-biotechnology platforms, material characterization must be linked directly to biological and processing performance. Standard analyses such as powder X-ray diffraction, Fourier-transform infrared spectroscopy, electron microscopy, dynamic light scattering, zeta potential, thermogravimetric analysis, and nitrogen adsorption are useful for confirming framework formation, morphology, colloidal behavior, thermal stability, and porosity [103,104]. In food and probiotic systems, these measurements should be complemented by simulated digestion studies, metal and linker release analysis, cytocompatibility testing, microbial viability, membrane integrity, metabolic activity, storage stability, and release kinetics.

Evaluation metrics should reflect the intended application. For probiotic delivery, relevant outcomes include survival during encapsulation, drying, storage, gastric and bile exposure, intestinal release, and recovery of metabolic function [105]. For precision fermentation, key parameters include enzyme activity, microbial productivity, substrate conversion, product yield, operational stability, reusability, mass transfer, and product recovery [106].

MOF-based platforms should also be compared with established food-grade materials such as alginate, chitosan, proteins, starch, pectin, lipids, and hydrogels, which already offer practical advantages in encapsulation and processing. MOFs are most likely to provide meaningful value when they enable functions that conventional matrices cannot easily achieve, including tunable degradation, selective metabolite adsorption, enzyme stabilization, responsive release, or integrated delivery and separation [107]. Future studies should report synthesis conditions, precursor concentrations, purification steps, residual components, encapsulation efficiency, food-matrix behavior, release profiles, and statistical analysis. Establishing these structure–function relationships will be essential for translating MOF-enabled systems into probiotic delivery, precision fermentation, and circular food technologies.

## 4. Metal–Organic Frameworks for Probiotic Delivery and Functional Food Applications

### 4.1. Delivery Barriers in Probiotic-Based Food Systems

The development of probiotic-based functional foods requires the preservation of microbial viability and functionality throughout processing, storage, ingestion, gastrointestinal transit, and release at the intended site of action. Probiotics are defined as live microorganisms that provide health benefits when administered in adequate amounts [108]. However, viability alone does not ensure probiotic efficacy, as cells must also retain stress tolerance, metabolic activity, adhesion potential, antagonistic effects against undesirable microorganisms, and the ability to produce beneficial metabolites under relevant food or physiological conditions [109].

Probiotic cells are exposed to multiple stressors during industrial processing and storage, including oxygen, heat, osmotic pressure, shear, dehydration, freeze-drying, spray-drying, acidic food environments, oxidative damage, moisture migration, and nutrient depletion [110]. After ingestion, they must further tolerate gastric acidity, digestive enzymes, bile salts, and competition with resident gut microorganisms before exerting functional effects [111]. These barriers are particularly important for sensitive strains, such as *Bifidobacterium* species, although tolerance varies substantially by strain origin and adaptation history [112].

**Figure 4:**
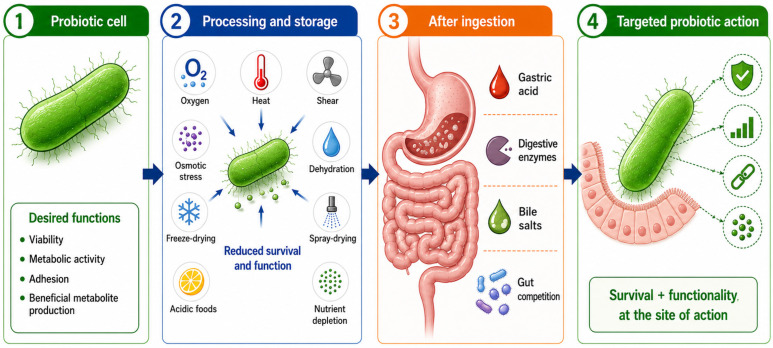
Delivery process and barriers in probiotic-based food systems.

Probiotic cells must maintain viability and functional activity across multiple food-processing, storage, and gastrointestinal barriers before reaching the intended site of action. These major barriers and their effects on probiotic survival and functionality are summarized in Figure 4.

Encapsulation systems based on alginate, chitosan, starch, pectin, gelatin, whey proteins, cellulose derivatives, lipids, and hydrogels have improved probiotic protection in many food matrices [113]. A report by Nguyen et al. (2026) confirmed that encapsulating oregano essential oil with Ag-doped ZIF-8 and hyaluronic acid significantly enhanced antimicrobial efficacy by improving stability, retention, and controlled release of active compounds, resulting in greater antibacterial activity than non-encapsulated formulations and conventional chlorine treatment [114]. Nevertheless, conventional matrices may suffer from weak mechanical stability, uncontrolled swelling, rapid diffusion of protons or bile salts, and limited responsiveness to gastrointestinal or food-processing conditions [115]. MOFs offer a complementary strategy because their framework composition, surface chemistry, degradation behavior, and molecular transport properties can be designed with greater structural precision, enabling more controlled probiotic protection and release than many traditional encapsulation materials [116]. This tunability should not, however, be interpreted as evidence that MOFs are intrinsically superior to established encapsulation materials. Their performance depends on framework composition, coating uniformity, residual precursors, degradation products, and interactions with the surrounding food matrix.

A major limitation in the current literature is the reliance on simplified buffer experiments, short exposure periods, and survival measurements without sufficiently rigorous controls. Many studies do not compare MOF-coated cells with uncoated cells, conventional encapsulation systems, unloaded MOF matrices, or individual metal and ligand precursors under identical conditions. Consequently, improved survival may be attributed to the MOF architecture even when it partly results from changes in pH, ionic strength, nutrient availability, aggregation, or residual synthesis components. Future studies should therefore incorporate matrix-only controls, precursor controls, matched environmental conditions, and post-release functional measurements. Evaluation in realistic food matrices and simulated gastrointestinal systems is also needed to determine whether protection observed in laboratory buffers is maintained in practical applications.

### 4.2. MOF-Based Encapsulation and Cytoprotection of Probiotic Cells

MOF-based probiotic delivery is conceptually different from conventional molecular encapsulation. Because microbial cells are larger than MOF micropores, the framework generally forms an external coating or mineralized matrix around the cell rather than encapsulating the cell inside internal pores [117]. This architecture can create an artificial protective layer that shields microorganisms from environmental stress while permitting diffusion of small molecules required for cell maintenance. The development of biomimetic MOF mineralization has been central to this area. Early work demonstrated that MOFs could be grown around biomacromolecules under mild conditions, providing enhanced protection against denaturation and degradation [118]. This strategy was later extended to living cells, where ZIF-8 coatings functioned as cytoprotective exoskeletons capable of maintaining cell viability while restricting cell division and inducing a reversible pseudo-dormant state [119].

**Figure 5:**
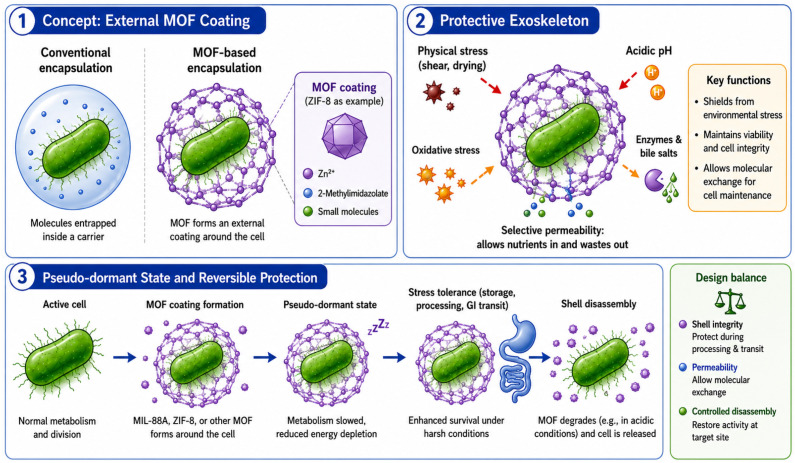
Mechanisms of MOF-Mediated Cytoprotection and Controlled Release of Probiotic Cells.

For probiotic applications, this pseudo-dormant behavior may be valuable if it reduces metabolic exhaustion during storage and improves survival under stress. However, the protective effect must be reversible because probiotic function ultimately requires reactivation after release. MOF coatings therefore need to balance three competing requirements: sufficient shell integrity to protect cells during processing and gastrointestinal transit, adequate permeability to allow molecular exchange, and controlled disassembly to restore cell activity at the target site [120]. ZIF-8 has been widely used as a model system because it can form rapidly under mild conditions and degrade in response to acidic environments [121]. Nevertheless, direct translation of ZIF-8 into food applications remains limited by concerns regarding zinc and imidazolate exposure, framework residues, and long-term dietary safety [122]. The key principles of MOF-based probiotic encapsulation are illustrated in Figure 5, highlighting the formation of protective exoskeletons around microbial cells, the induction of a reversible pseudo-dormant state during storage and gastrointestinal transit, and the controlled reactivation of probiotic function at the target site.

Recent studies have begun to expand the material library toward more biocompatible frameworks. A notable example is the encapsulation of *Lactiplantibacillus plantarum* 299v in a nanocrystalline MIL-88A matrix composed of iron and fumaric acid [123]. This approach is important because it moves MOF-assisted bacterial encapsulation beyond model organisms and toward probiotic-relevant strains and more food-compatible components. Iron and fumarate are more familiar within nutritional and food contexts than many synthetic MOF constituents, although their assembled framework, degradation behavior, and exposure profile still require systematic safety assessment [124]. Such work suggests that probiotic delivery may benefit from MOFs designed specifically for food compatibility rather than from direct adaptation of biomedical MOFs.

### 4.3. Protection Against Processing and Gastrointestinal Stress

The most immediate function of MOF-based probiotic coating is protection against environmental and gastrointestinal stress. MOF shells may reduce direct contact between cells and acidic media, reactive oxygen species, bile salts, digestive enzymes, and antimicrobial compounds [125]. The protective mechanism depends on shell thickness, crystallinity, porosity, surface charge, and degradation behavior. Thin, porous coatings may allow sufficient nutrient and metabolite exchange, while thicker or denser coatings may provide greater stress resistance but reduce metabolic activity or delay reactivation [126]. In this respect, MOF-based encapsulation requires optimization at the cell-material interface rather than simple maximization of coating density.

Gastric protection is especially important because gastric acidity can rapidly reduce probiotic viability. Conventional alginate or protein-based capsules often improve survival, but their protection can be compromised by proton diffusion, matrix swelling, or premature disintegration. MOFs may offer stronger control over diffusion and degradation because their crystalline networks and coordination chemistry can be tuned at the molecular level [127]. However, the degradation profile must align with the intended biological route. A framework that dissolves too quickly in gastric acid may release cells prematurely, whereas one that remains stable too long may delay probiotic activity in the intestine. For this reason, multilayer designs that combine MOFs with biopolymers may be more effective than single-component MOF coatings for oral probiotic delivery [128].

Bile tolerance and intestinal release are equally relevant. Bile salts can disrupt bacterial membranes and reduce survival after gastric transit [129]. MOF coatings may reduce bile-cell contact, but release must occur under conditions that permit recovery of metabolic function. Enteric biopolymer layers, pH-responsive linkers, or degradable food-compatible frameworks may allow sequential protection and release. For example, a hybrid system could use an outer polysaccharide layer to reduce gastric degradation and an inner MOF shell to provide structural cytoprotection and controlled exposure to intestinal conditions [130]. Such architectures may be especially useful for sensitive probiotic strains or non-dairy food matrices in which cells lack the protective buffering effects of milk proteins and fat.

### 4.4. Hybrid MOF–Biopolymer Platforms for Food Matrices

Hybridization with food-grade biopolymers is one of the most realistic strategies for adapting MOFs to probiotic foods. Natural polymers such as alginate, chitosan, pectin, starch, gelatin, cellulose derivatives, and whey proteins are already widely used for probiotic encapsulation and oral delivery [131]. These materials provide hydration, film formation, gelation, acid buffering, mucoadhesion, and compatibility with food processing. When combined with MOFs, they can reduce direct biological exposure to framework components, improve dispersion, adjust surface charge, and provide additional diffusion barriers [132].

Alginate–MOF systems may be particularly useful because alginate forms gels under mild ionic conditions and has been widely used for probiotic microencapsulation [133]. However, alginate beads can be mechanically weak and vulnerable to destabilization in chelating environments. Incorporating MOFs into alginate-based matrices may improve structural reinforcement, molecular adsorption, or controlled release. The Alginate@ZIF-8/Moringa oleifera nano-biocomposite demonstrated excellent performance for methylene blue removal from aqueous solutions, achieving a maximum adsorption capacity of 297.84 mg/g and removal efficiency exceeding 98.5% [134]. Adsorption followed pseudo-second-order kinetics and the Langmuir isotherm model, indicating monolayer chemisorption. The process was spontaneous and endothermic, while the composite retained over 90% removal efficiency after five regeneration cycles, highlighting its potential as a sustainable and reusable adsorbent for wastewater treatment.

On the other hand, chitosan can provide a cationic coating that strengthens alginate capsules and improves acid resistance, but its antimicrobial properties require careful control to avoid reducing probiotic viability [135]. MOF–chitosan or MOF–alginate–chitosan systems may allow multilayer protection in which each component contributes a distinct function [136].

Protein-based matrices offer another important route for food integration. Whey proteins, caseins, gelatin, and plant proteins can buffer gastric acidity, interact with microbial surfaces, and improve sensory compatibility in functional foods [137]. Protein–MOF composites may be useful for dairy and plant-based fermented products because they can integrate delivery function with nutritional and textural properties [138]. Starch, pectin, and cellulose derivatives may support release in the lower gastrointestinal tract due to their resistance to upper gastrointestinal digestion and their fermentation by gut microbiota [138]. These polymers could be combined with MOFs to develop synbiotic systems in which probiotics are protected together with fermentable substrates or bioactive compounds.

The design of MOF–biopolymer systems must consider food-matrix complexity. Proteins, salts, organic acids, phenolics, emulsifiers, and lipids may adsorb to MOF surfaces, alter particle aggregation, modify degradation, or compete with biological cargo [139]. These interactions may improve biocompatibility by forming protective coronas, but they may also reduce pore accessibility or modify release kinetics.

### 4.5. Strain-Specific Functionality and Biological Performance

The response of probiotics to MOF encapsulation is expected to be highly strain-dependent. Cell envelope composition, surface charge, hydrophobicity, stress-response pathways, oxygen tolerance, acid resistance, bile tolerance, and metabolic activity vary substantially among probiotic microorganisms [140]. Gram-positive lactic acid bacteria possess thick peptidoglycan layers and teichoic acids, which may promote coordination-mediated nucleation of MOFs [141]. *Bifidobacterium* species are often more oxygen-sensitive and may require stronger protection against oxidative stress [142]. Probiotic yeasts such as *Saccharomyces boulardii* have different cell wall structures dominated by glucans and mannoproteins, which may produce distinct MOF nucleation and coating behaviors [143].

**Figure 6:**
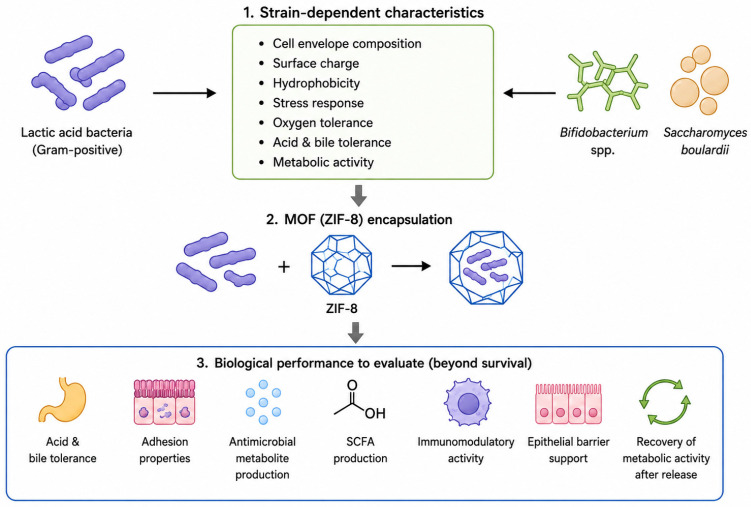
Strain-specific considerations for evaluating MOF-based probiotic encapsulation systems.

Because probiotic benefits are strain-specific, MOF, specifically ZIF-8, encapsulation should be evaluated beyond general survival. Important functional endpoints include acid and bile tolerance, adhesion-related properties, antimicrobial metabolite production, short-chain fatty acid formation, immunomodulatory activity, epithelial barrier support, and recovery of metabolic activity after release [144]. A coating that increases survival but suppresses functional traits may not improve probiotic efficacy [145]. Similarly, a system that preserves viability in buffer may fail in a real food matrix or during storage. Lu et al. (2023) developed a mixed-ligand ZIF-8 (ZIF-8-IM) that was engineered to enhance the separation of C6 alkane isomers [146]. Ligand exchange modified pore dimensions, significantly improving adsorption and diffusion of mono-branched 3-methylpentane relative to di-branched 2,3-dimethylbutane. A high-quality evaluation should therefore link MOF structure to both viability and strain-specific biological function. As illustrated in Figure 6, comprehensive evaluation of MOF-encapsulated probiotics should integrate strain-specific physiological characteristics with post-release biological functionality rather than relying solely on viability measurements.

The possibility of temporary metabolic suppression during MOF coating requires careful interpretation. In living-cell MOF studies, cytoprotective shells can restrict cell division while maintaining viability [146]. For storage, this may be advantageous because reduced proliferation can limit nutrient consumption and acid accumulation. For delivery, however, cells must recover after shell removal or degradation. Future probiotic studies should therefore measure not only immediate colony-forming units but also post-release growth kinetics, metabolic activity, membrane integrity, and production of functional metabolites.

### 4.6. MOF-Enabled Synbiotic and Functional Food Systems

MOF-based delivery platforms may also support the development of synbiotic and multifunctional foods. Synbiotic systems combine probiotics with substrates selectively utilized by beneficial microorganisms [147]. MOFs could contribute to such systems by protecting probiotics while co-delivering prebiotics, polyphenols, vitamins, minerals, antioxidants, enzymes, or antimicrobial compounds [148]. Their tunable porosity and surface chemistry may allow small bioactives to be adsorbed within the framework or associated with external polymer layers, creating delivery systems with coordinated release.

In fermented foods, MOF-assisted delivery could be used not only to protect probiotic cultures but also to stabilize starter cultures, regulate metabolite release, or support post-fermentation survival. Products such as yogurt, kefir, kombucha, fermented plant-based beverages, fermented cereals, and probiotic powders may benefit from encapsulation systems that improve shelf life without compromising sensory quality [149]. MOF-based systems may also be useful in non-dairy probiotic foods, where acidity, oxygen exposure, and limited buffering capacity often reduce cell survival [150].

The use of MOFs in functional foods must be guided by translational realism. Materials intended for consumption should be prepared from safe precursors, synthesized under food-compatible conditions, purified thoroughly, and evaluated under simulated digestion and realistic intake scenarios [151]. Sensory impact, color, flavor interaction, mouthfeel, labeling, consumer perception, cost, and regulatory classification will strongly influence adoption. Therefore, MOF-based probiotic delivery should not be framed simply as a replacement for conventional encapsulation. Its strongest potential lies in applications where conventional matrices cannot provide sufficient control over protection, release, co-delivery, or multifunctional performance.

### 4.7. Translational Outlook for MOF-Based Probiotic Delivery

The development of MOF-based probiotic delivery systems remains at an early stage, yet the field is gradually progressing from proof-of-concept studies toward biologically relevant and application-oriented designs. Future advances are expected to prioritize biocompatible frameworks, food-compatible or nutritionally relevant building blocks, aqueous and environmentally benign synthesis routes, and hybrid systems incorporating established food-grade polymers. Equally important is the evaluation of these materials under realistic food matrices and simulated gastrointestinal conditions to better reflect their practical performance.

Recent work has begun to demonstrate the feasibility of this transition. For example, the biocompatible iron–fumarate MOF MIL-88A was successfully employed to encapsulate *Lactiplantibacillus plantarum* 299v through a simple one-pot synthesis, producing a nanocrystalline coating that significantly enhanced bacterial stability. Encapsulation increased probiotic viability by approximately 2.2–2.7-fold during saline storage while providing improved resistance to lysozyme and pepsin, suggesting that food-compatible MOFs can effectively protect probiotics during oral and gastric transit [152]. These findings highlight the potential of alternative MOF chemistries beyond conventional zinc-based systems.

Although ZIF-8 will likely remain an important model platform because of its well-defined structure and ease of synthesis, broader application in food systems will require frameworks with improved biocompatibility, predictable degradation, and greater regulatory acceptability [152]. The continued development of iron-, calcium-, magnesium-, or other nutritionally relevant MOFs may therefore provide more practical pathways for food and probiotic applications.

Future research should move beyond demonstrating probiotic survival and adopt standardized evaluation frameworks that integrate both materials science and biological functionality. Comprehensive characterization of framework composition, shell morphology, residual precursor content, metal release, degradation kinetics, and stability in food matrices should be accompanied by assessments of probiotic viability, gastrointestinal stress resistance, post-release growth, colonization potential, and functional metabolite production. Such evaluation will enable meaningful comparisons between MOF-based delivery systems and established encapsulation platforms, including alginate, chitosan, proteins, lipids, and hydrogels, thereby clarifying when MOFs provide genuine functional advantages rather than incremental material novelty. If these challenges can be addressed, MOF-enabled encapsulation has the potential to become a versatile platform for next-generation probiotic foods, synbiotic formulations, and precision nutrition.

## 5. Metal–Organic Frameworks for Precision Fermentation and Bioprocess Intensification

### 5.1. Relevance of MOFs to Precision Fermentation

Precision fermentation has emerged as an important food-biotechnology platform for producing targeted ingredients through microorganisms programmed or selected to synthesize specific compounds, including proteins, enzymes, lipids, vitamins, pigments, flavor molecules, sweeteners, organic acids, and bioactive metabolites [153]. Unlike traditional fermentation, which often relies on complex microbial consortia and broad metabolic transformation of raw substrates, precision fermentation is designed around defined microbial cell factories, controlled process parameters, and product-specific optimization [154]. This approach is gaining attention for producing animal-free dairy proteins, egg proteins, heme proteins, specialty fats, enzymes, aroma compounds, and nutritionally relevant micronutrients [155]. However, the scalability of precision fermentation depends on overcoming persistent challenges related to cell stress, product inhibition, substrate utilization, oxygen and mass transfer, biocatalyst instability, downstream separation, and process cost [156].

MOFs are relevant to these challenges because their high surface area, tunable pore chemistry, molecular selectivity, and functionalizable interfaces can be adapted to stabilize biocatalysts, immobilize microorganisms, adsorb inhibitory compounds, regulate microenvironments, and support product recovery [157]. In this context, MOFs should not be viewed only as passive carriers but as functional scaffolds that can influence the local chemical environment of enzymes, cells, substrates, and metabolites. Their use in precision fermentation is still less mature than their use in gas separation, catalysis, drug delivery, or enzyme immobilization, but the design principles developed in those fields provide a foundation for food-bioprocessing applications [158].

The strongest near-term opportunities are likely to arise in three areas. First, MOFs can stabilize enzymes used in upstream substrate conversion, cofactor regeneration, and post-fermentation product modification [159]. Second, MOFs and MOF-based composites can provide immobilization platforms for microbial cells, potentially enabling repeated-batch or continuous fermentation with improved operational stability [160]. Third, MOFs may assist downstream processing by selectively adsorbing target metabolites or removing inhibitory compounds from fermentation broths [161]. These functions align closely with precision fermentation because process efficiency depends not only on the genetic capability of the microbial strain but also on the materials and separation systems that sustain productivity throughout cultivation and recovery.

### 5.2. Enzyme@MOF Biocomposites for Fermentation-Associated Biocatalysis

Enzymes play essential roles across fermentation processes, including starch and cellulose hydrolysis, lactose conversion, protein modification, lipid transformation, flavor generation, phenolic conversion, and synthesis of high-value food ingredients [162]. Free enzymes are often limited by poor stability under changing pH, temperature, ionic strength, solvent composition, and product accumulation. Immobilization can improve enzyme stability and reusability, but conventional supports may suffer from low loading capacity, uncontrolled enzyme orientation, leaching, restricted mass transfer, or loss of catalytic activity [163]. MOFs provide a promising platform because their pore structure, surface functionality, and coordination chemistry can be designed to interact with enzymes through adsorption, encapsulation, covalent attachment, pore infiltration, or biomimetic mineralization [163].

Enzyme@MOF systems can improve thermal stability, pH tolerance, storage stability, solvent resistance, and operational reusability by physically confining enzymes and reducing conformational mobility [164]. Horseradish peroxidase (HRP) encapsulated within a ZIF-8/graphene oxide (GO) hybrid coating exhibited substantially enhanced stability under harsh conditions, including high temperatures, proteolytic enzymes, and chemical stressors [165]. The composite improved substrate affinity while preserving catalytic activity. Additionally, the graphene-containing system enabled DNA-mediated regulation of peroxidase activity and supported sensitive colorimetric biosensing, demonstrating the potential of MOF-based enzyme encapsulation to improve enzyme durability and expand applications in biocatalysis, biosensing, and industrial biotechnology.

Biomimetic mineralization is especially important because enzymes can be incorporated during framework formation under relatively mild conditions, producing protective shells that reduce denaturation and proteolysis [166]. Studies on protein@ZIF-8 and related biocomposites have shown that MOF matrices can preserve biomolecular activity while improving resistance to environmental stress [167]. These findings are highly relevant to precision fermentation because many food-relevant enzymatic reactions occur in complex aqueous media containing salts, peptides, sugars, organic acids, and inhibitory metabolites.

The translation of enzyme@MOF platforms into fermentation should be guided by reaction-specific design. Hydrolytic enzymes involved in biomass pretreatment require sufficient substrate access and tolerance to variable feedstock composition [168]. Enzymes used for flavor generation or post-fermentation modification require high selectivity and minimal off-flavor formation. Cofactor-dependent enzymes may suggest MOF designs that support cofactor retention, diffusion, or regeneration [169]. Because many industrial enzymes are larger than MOF micropores, mesoporous MOFs, defect-engineered frameworks, hierarchical MOF composites, and surface-confined immobilization strategies may be more suitable than simple microporous encapsulation. The value of MOFs will be greatest when they provide stability and reusability without imposing mass-transfer limitations that reduce overall productivity.

### 5.3. MOF-Assisted Microbial Immobilization and Cell Factory Stabilization

Microbial immobilization is widely used to improve cell retention, enable repeated use of biomass, increase resistance to environmental stress, and support continuous or semi-continuous bioprocessing [170,171]. In precision fermentation, immobilized cells may reduce inoculum cost, improve volumetric productivity, simplify separation of biomass from products, and enhance resilience under high-cell-density cultivation. Conventional immobilization systems include alginate beads, polyacrylamide gels, cellulose matrices, chitosan, silica, biochar, and polymeric supports [172]. These materials are useful but may lack molecular selectivity, tunable microenvironments, or integrated product-separation functions.

MOFs and MOF-based composites may expand the functionality of microbial immobilization platforms. Their porous and chemically tunable structures can provide local adsorption sites for nutrients, cofactors, metal ions, or inhibitory compounds, while their surfaces can be modified to promote or limit cell adhesion [173]. For living microorganisms, direct MOF encapsulation must preserve membrane integrity, nutrient diffusion, metabolic function, and the ability to recover or proliferate when appropriate [174]. Living-cell MOF coatings have demonstrated that protective mineralized shells can shield cells and modulate activity, providing a conceptual basis for immobilized microbial cell factories [175]. However, precision fermentation may require different design criteria than probiotic delivery. Rather than promoting release after gastrointestinal transit, immobilized fermentation systems may require long-term stability, repeated operation, and controlled mass transfer between immobilized cells and the surrounding medium.

The effectiveness of MOF-assisted microbial immobilization depends strongly on the physiological characteristics of the host microorganism. Yeasts, filamentous fungi, lactic acid bacteria, *Bacillus* spp., *Corynebacterium* spp., and engineered *Escherichia coli* differ substantially in cell-envelope structure, morphology, oxygen requirements, secretion behavior, and tolerance to metal ions or organic ligands [176,177]. As a result, the interactions between microbial cells and MOF materials can vary considerably, influencing framework nucleation, coating uniformity, cell viability, and metabolic activity. Importantly, a coating that enhances stress resistance may not necessarily improve bioprocess performance if it restricts nutrient transport, cell growth, or product secretion. Consequently, the success of MOF-assisted immobilization should be evaluated using process-relevant metrics, including substrate utilization, product yield, productivity, secretion efficiency, operational stability, and cell viability, rather than coating formation alone [178].

From a practical perspective, hybrid MOF–biopolymer systems may offer greater potential for food fermentation than free MOF particles. Biopolymers such as alginate, chitosan, cellulose, gelatin, and proteins provide established biocompatibility and mechanical stability, while MOFs can contribute selective adsorption, catalytic activity, and microenvironmental control [179,180]. The combination of these properties could enable multifunctional immobilization platforms capable of retaining microbial cells, stabilizing enzymes, and mitigating product inhibition within a single system. Such approaches may be particularly valuable in fermentation processes where cell reuse, metabolite accumulation, or downstream separation represent major constraints on process efficiency and economic viability.

### 5.4. MOFs for Product Inhibition Control and Metabolite Recovery

Product inhibition is a common limitation in fermentation. Accumulation of organic acids, alcohols, solvents, phenolics, aldehydes, fatty acids, or secreted proteins can reduce cell growth, impair enzyme activity, alter membrane integrity, and limit final product titer [181]. Precision fermentation is especially sensitive to these effects because high productivity often requires high product concentration and controlled metabolic flux. In situ product removal can improve fermentation performance by reducing inhibitory concentrations while simplifying downstream recovery [182].

MOFs are attractive for in situ or downstream separation because their pore environments can be tuned for polarity, size selectivity, charge interaction, hydrophobicity, and coordination affinity [183]. In fermentation broths, potential targets include volatile fatty acids, organic acids, aroma compounds, pigments, phenolics, peptides, enzymes, and other high-value metabolites [184]. MOFs may also remove inhibitory compounds generated during lignocellulosic or food-waste hydrolysis, including furans, phenolics, and organic acids, thereby improving microbial fermentation of low-cost substrates [185]. Compared with nonselective adsorbents, MOFs offer the possibility of designing binding sites for specific functional groups, although fouling by proteins, salts, and polysaccharides remains a significant concern. The main potential applications and design considerations of MOFs for product inhibition control and metabolite recovery are summarized in Table 1.

The application of MOFs in metabolite recovery should consider broth complexity. Fermentation media contain cells, extracellular polymeric substances, proteins, peptides, minerals, surfactants, antifoams, and residual substrates that can compete for adsorption sites or block pores [190]. MOFs that perform well in model solutions may lose selectivity in real broths. Water stability is also essential because many frameworks degrade in aqueous or acidic environments. Iron-, zirconium-, and selected zeolitic imidazolate frameworks may provide greater stability, but their regeneration, leaching, and food-contact safety must be assessed [191,192]. For food applications, adsorbents should also avoid product contamination and must be separable from the final ingredient stream.

MOF-based in situ product removal may be particularly useful when integrated into immobilized or membrane-based systems. Rather than adding free nanoparticles to fermentation broth, MOFs could be incorporated into fixed beds, membranes, beads, magnetic composites, or hydrogel supports that allow product capture while minimizing direct particle contamination [193,194]. This configuration may improve process control and regulatory feasibility. In downstream processing, MOF columns or composite membranes could selectively concentrate target metabolites before polishing steps, potentially reducing solvent use and energy demand [195]. The feasibility of these approaches will depend on adsorption capacity, selectivity, regeneration efficiency, mechanical stability, and compatibility with food-grade processing.

### 5.5. Process Intensification Through MOF-Integrated Fermentation Systems

Process intensification seeks to improve productivity, reduce resource consumption, and enhance product recovery through the integration of reaction and separation processes [196]. In this context, MOFs are attractive because they can provide multiple functions within a single platform, including enzyme stabilization, cell immobilization, inhibitor removal, and metabolite recovery [158]. Such multifunctionality is particularly relevant to precision fermentation, where overall process performance depends on both biological and engineering factors.

Several implementation strategies have been proposed, including enzyme@MOF composites for substrate conversion, MOF–biopolymer beads for microbial immobilization, MOF membranes for selective separations, magnetic MOF composites for material recovery, and fixed-bed MOF adsorbents for product capture or inhibitor removal [197,198]. However, their practical deployment must balance mass transfer, fouling, regeneration, sterility, cost, and regulatory considerations.

Future integration of MOFs with precision fermentation may also benefit from data-driven design. Machine learning and high-throughput bioprocess datasets could help identify MOFs with suitable pore structures, surface properties, stability, and adsorption behavior for specific fermentation objectives. Nevertheless, predictive models will require standardized datasets generated under realistic fermentation conditions rather than simplified laboratory systems.

### 5.6. Translational Considerations for Food-Grade Fermentation Applications

The use of MOFs in precision fermentation will depend on whether their technical advantages justify their complexity, cost, and regulatory burden. For edible products, direct contact between MOFs and the final food ingredient raises questions regarding residual particles, metal release, ligand migration, and toxicological evaluation [199]. For processing aids or immobilized reactor materials, regulatory concerns may be reduced if MOFs are retained and analytically shown not to contaminate the final product [200]. Therefore, immobilized formats, composite membranes, fixed-bed adsorbents, and recoverable particles may be more realistic near-term applications than direct incorporation of free MOFs into food fermentation broths.

A food-grade precision fermentation platform using MOFs should prioritize aqueous synthesis, safe metal nodes and linkers, framework stability under process conditions, efficient regeneration, and validated absence of harmful residues in the product stream [201]. The performance metrics should include not only enzyme activity or adsorption capacity but also product titer, yield, productivity, purity, sensory quality, process economics, and environmental footprint. MOFs may be most valuable in high-value ingredient production, where improved stability, selectivity, or recovery can offset material cost. Their use in bulk commodity fermentation will require stronger evidence of scalability and cost-effectiveness [202].

In summary, MOFs offer a versatile materials platform for precision fermentation by supporting enzyme stabilization, microbial immobilization, inhibition control, metabolite recovery, and process integration. Their strongest potential lies in systems where molecular selectivity, reusable biocatalysis, and integrated separation can improve productivity beyond what conventional fermentation supports can achieve. Future research should move from model reactions toward food-relevant organisms, realistic fermentation broths, scalable composite formats, and complete techno-functional evaluation.

## 6. Metal–Organic Frameworks for Circular Food Systems and Fermentation Waste Valorization

### 6.1. Connecting MOF Functionality with Circular Food-System Design

Circular food systems aim to reduce resource loss by converting food-processing residues, fermentation by-products, and nutrient-rich effluents into valuable ingredients, chemicals, materials, fertilizers, or energy carriers [203]. This concept aligns closely with fermentation biotechnology because many food residues are rich in carbohydrates, proteins, lipids, minerals, fiber, and bioactive compounds that microorganisms or enzymes can transform into value-added products [204]. Examples include converting fruit pomace into organic acids or phenolic-rich extracts, whey permeate into lactic acid or microbial biomass, spent grain into fermentable sugars, and vegetable residues into volatile fatty acids or microbial protein [203,205]. Despite this potential, practical valorization is often constrained by dilute product streams, heterogeneous feedstock composition, inhibitory compounds, complex separation requirements, and variable microbial performance [206].

MOFs are relevant to circular food systems because they can contribute functions that are difficult to achieve using fermentation alone. Their tunable porosity, surface chemistry, metal nodes, and linker environments can support selective adsorption, catalytic transformation, nutrient recovery, enzyme stabilization, and removal of fermentation inhibitors [207,208]. For example, a MOF-based adsorbent could be used after acidogenic fermentation to concentrate acetic, propionic, or butyric acid from a dilute broth [209], while an enzyme@MOF composite could hydrolyze starch- or cellulose-rich residues before microbial conversion [209]. Similarly, MOF-containing membranes or beads could remove phenolic inhibitors from lignocellulosic hydrolysates, allowing yeasts or lactic acid bacteria to ferment sugars more efficiently [189]. These examples illustrate how MOFs may function as enabling materials within integrated food-waste biorefineries rather than as standalone waste-treatment tools. The potential integration points of MOFs within circular food-waste biorefineries are summarized in Table 2.

The strongest value of MOFs in circular food systems is likely to arise from integration with existing fermentation processes. Food residues are rarely converted efficiently through a single step. A realistic process may involve pretreatment, enzymatic hydrolysis, microbial fermentation, product recovery, nutrient recycling, and treatment of the remaining effluent [215]. MOFs could be positioned at different points in this chain: as catalysts during pretreatment, immobilization supports during fermentation, adsorbents during product recovery, or nutrient-capture materials during wastewater polishing. Such integration is especially important because many waste-derived products, including volatile fatty acids, amino acids, organic acids, pigments, and phenolics, are produced at relatively low concentrations and require selective recovery to become economically viable [216,217].

### 6.2. Valorization of Fermentation Residues and Food-Processing Side Streams

Fermentation generates several types of side streams, including spent microbial biomass, residual substrates, organic acids, soluble proteins, peptides, minerals, cell debris, and wastewater [215,218]. In brewing, spent grain and spent yeast contain proteins, fibers, minerals, and bioactive compounds that can be recovered or biologically converted [219]. In dairy processing, whey and whey permeate are rich in lactose and can serve as substrates for lactic acid, ethanol, microbial oil, or single-cell protein production [220]. Fruit and vegetable processing generates pomace, peels, seeds, and pulp residues that contain fermentable sugars, pectin, cellulose, polyphenols, pigments, and organic acids [221,222]. Precision fermentation also produces side streams containing unused nutrients, salts, secreted metabolites, and microbial biomass that may be recycled if properly separated and stabilized [215,223].

MOFs may support the valorization of these streams in several specific ways. First, enzyme@MOF systems can improve the hydrolysis of complex residues before fermentation. Cellulases, amylases, proteases, lipases, pectinases, and β-glucosidases are commonly used to release fermentable sugars, peptides, fatty acids, and phenolics from food by-products [224,225]. Immobilizing these enzymes in or on MOFs can improve their thermal stability, pH tolerance, and reusability, which is important when processing heterogeneous residues such as apple pomace, citrus peel, soybean okara, or brewer’s spent grain [226]. Second, MOFs can remove inhibitory molecules that reduce microbial conversion. Lignocellulosic and thermally processed residues may contain furfural, 5-hydroxymethylfurfural, phenolics, and weak acids that inhibit yeast and bacterial metabolism [227]. MOFs with hydrophobic pores, open metal sites, or functionalized linkers could be designed to capture these inhibitors before or during fermentation.

Third, MOFs may improve the recovery of target products from dilute fermentation broths. Volatile fatty acids generated through anaerobic fermentation of food waste are valuable precursors for bioplastics, biofuels, microbial lipids, and chain-elongated products [228]. However, their recovery is challenging because they are produced in water, often as mixed acids, and may coexist with salts, proteins, and residual sugars. Adsorption-based recovery using porous materials has been investigated as a lower-energy alternative to distillation or solvent extraction [229]. MOFs could extend this strategy by providing tunable binding sites for carboxylates, although they must be stable in acidic and saline fermentation broths. For example, zirconium-based MOFs, iron-based MOFs, or MOF-polymer composites may be more suitable than water-labile frameworks when repeated adsorption and desorption cycles are required [42,230].

Despite encouraging progress, an important experimental gap remains in the current literature. Many studies evaluate probiotic survival or antimicrobial performance without performing sufficient control experiments to distinguish the intrinsic behavior of the MOF matrix from the effects of the encapsulated biological cargo or loaded bioactive compounds. Because some MOFs exhibit catalytic activity, redox behavior, or metal-ion-mediated antimicrobial effects, it is important to evaluate the framework independently under identical experimental conditions. Appropriate controls should determine whether the observed biological response arises from the protective encapsulation function, the catalytic properties of the MOF itself, or synergistic interactions between the framework and the encapsulated material. Such systematic comparisons would strengthen mechanistic understanding, improve reproducibility, and support the rational design of food-grade MOF delivery systems.

### 6.3. MOFs for Nutrient Recovery from Food and Fermentation Effluents

Food and fermentation effluents often contain nitrogen, phosphorus, potassium, organic carbon, and trace minerals that can contribute to eutrophication if discharged without recovery [231]. At the same time, these nutrients are valuable resources for fertilizer production, microbial cultivation, and circular agriculture. Conventional nutrient recovery approaches include struvite precipitation, ammonia stripping, ion exchange, membrane separation, and adsorption [232]. MOFs may contribute to this area because their metal nodes and functionalized linkers can be designed to interact with phosphate, ammonium, nitrate, and organic nitrogen species [52,233]. Potential MOF-enabled nutrient recovery pathways from food and fermentation effluents are summarized in Table 3.

Phosphate recovery is a particularly relevant example. Phosphate can bind strongly to metal centers such as zirconium, lanthanum, iron, and aluminum through ligand exchange or inner-sphere complexation [237]. Zirconium-based MOFs and MOF-derived materials have been studied for phosphate adsorption because zirconium sites can show strong affinity toward phosphate groups [238]. In a food-system context, such materials could be used to recover phosphorus from fermentation wastewater, dairy effluents, or digestate streams after anaerobic treatment. The recovered phosphate could potentially be regenerated into a concentrated nutrient solution or incorporated into fertilizer formulations [239], although food-grade and environmental safety considerations would depend on the material composition and regeneration chemicals used.

Ammonium recovery represents another opportunity. Fermentation and anaerobic digestion streams may contain ammonium derived from protein degradation, yeast extract, peptone, or nitrogen-rich food residues [240]. Although zeolites and biochars are more established for ammonium adsorption, functionalized MOFs may offer higher tunability through charged sites, ion-exchange functionality, and pore-size control [241]. A practical example would be the polishing of a fermentation centrate after microbial biomass production, where ammonium could be captured and reused as nitrogen feed for another fermentation cycle. However, MOFs would need to compete with inexpensive materials such as zeolite, biochar, and ion-exchange resins; therefore, they are more likely to be justified when selective recovery, regeneration efficiency, or integration with other functions provides a clear advantage [242].

Nutrient recovery also has implications for precision fermentation. Many precision fermentation processes rely on refined nitrogen and phosphorus sources, which contribute to cost and environmental burden [243]. If nutrients from side streams can be recovered and purified, they may partially replace virgin inputs. For example, ammonium and phosphate recovered from food-processing wastewater could be reused in microbial media after quality control, while residual organic nitrogen from spent yeast could be enzymatically hydrolyzed into peptides or amino acids for microbial cultivation [244]. MOFs may assist in separating these nutrient fractions, removing inhibitory compounds, or stabilizing enzymes used in nutrient release.

### 6.4. MOF-Based Catalytic Conversion of Food-Derived Residues

Beyond adsorption, MOFs may contribute to circular food systems through catalytic conversion. Their metal nodes, organic linkers, and confined pore environments can provide catalytic sites for oxidation, reduction, hydrolysis, transesterification, and acid-base reactions [245]. In food-waste valorization, these reactions may be relevant for converting carbohydrates, lipids, phenolics, and organic acids into platform chemicals or functional ingredients. For example, carbohydrate-rich residues can be transformed into 5-hydroxymethylfurfural, levulinic acid, or lactic acid derivatives; lipid-rich residues can be converted into biodiesel or structured lipids; and phenolic-rich streams can be modified to improve antioxidant or antimicrobial functionality [246,247].

MOF-based catalysts may be particularly useful when selectivity is required. Unlike bulk acid or base catalysts, MOFs can provide defined active sites and microenvironments that influence substrate orientation and product distribution [248]. MOF-derived metal oxides or carbonaceous materials may also be used for catalytic upgrading of fermentation residues after thermal treatment [249]. For instance, spent microbial biomass or solid fermentation residues could be pyrolyzed into biochar-like materials, which may then be combined with MOF-derived catalysts for pollutant removal, nutrient adsorption, or bioenergy applications [250]. Although this approach moves beyond direct food use, it fits the circular-system objective of converting residues into functional materials.

**Figure 7:**
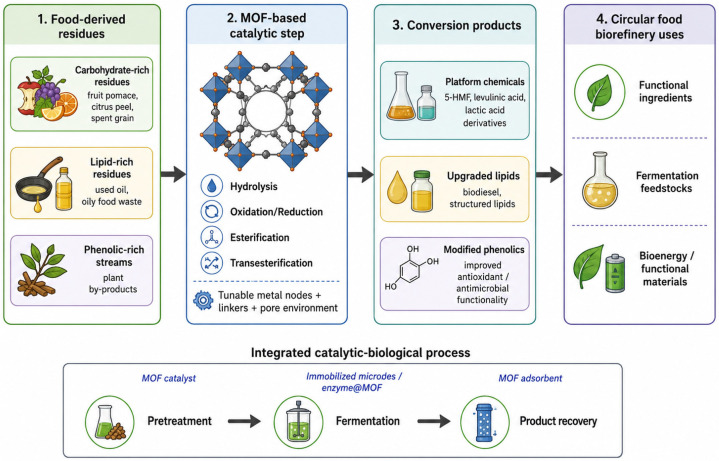
Schematic illustration of MOF-based catalytic conversion of food-derived residues into value-added products and their integration within circular food biorefineries.

In fermentation-based biorefineries, catalytic and biological steps may be combined. A MOF catalyst could pretreat a residue to release fermentable sugars, an immobilized microbial system could convert the sugars into organic acids or alcohols, and a MOF adsorbent could recover the product from the broth [37]. For example, citrus peel contains pectin, cellulose, hemicellulose, essential oils, and phenolics; a combined process might use enzymatic hydrolysis to release sugars, adsorption to remove limonene or phenolic inhibitors, and fermentation to produce lactic acid or volatile fatty acids [251,252]. MOFs could contribute to the adsorption or catalytic steps if their stability and selectivity are sufficient under the process conditions. Figure 7 illustrates how MOFs can support catalytic conversion, integrated fermentation workflows, and product recovery in circular food-system applications.

### 6.5. Integration with Fermentation-Based Biorefineries

The practical implementation of MOFs in circular food systems will depend largely on process configuration and material recoverability. Direct addition of free MOF nanoparticles into food-waste streams may complicate downstream separation and increase the risk of material carryover. Consequently, more practical approaches include MOF-coated beads, MOF-polymer membranes, fixed-bed adsorbents, magnetic MOF composites, and immobilized enzyme supports, which enable interaction with process streams while facilitating recovery and reuse [253]. For example, water-stable MOFs incorporated into packed columns may be used for volatile fatty acid recovery from fermentation broths, whereas magnetic MOF composites could be recovered after selective removal of phenolic inhibitors from hydrolysates [254].

The economic feasibility of MOF deployment should also be considered in relation to product value. Applications involving high-value compounds, such as flavors, pigments, bioactive molecules, specialty organic acids, and fermentation-derived proteins, may better justify the use of advanced MOF materials. In contrast, for bulk products, MOFs must demonstrate clear advantages in selectivity, regeneration, durability, or process efficiency compared with established technologies, including activated carbon, zeolites, membrane systems, and ion-exchange resins [233,255].

MOFs may also contribute to closed-loop bioprocessing by facilitating nutrient recovery, inhibitor removal, enzyme reuse, and metabolite separation [38]. Such functions could support the recycling of water, nutrients, and processing streams, thereby improving resource efficiency and reducing waste generation within circular food-production systems.

### 6.6. Sustainability and Translational Constraints

The environmental value of MOF-enabled valorization should be assessed across the full material life cycle. MOFs used in circular food systems should not create new sustainability burdens through solvent-intensive synthesis, toxic or rare metals, poor regeneration, or difficult disposal [256]. For this reason, circular applications should prioritize water-based synthesis, low-toxicity metal nodes, renewable linkers, efficient regeneration, and recoverable material formats.

Safety and regulatory considerations are equally important when valorized streams are intended for food, feed, fertilizer, or processing applications. Residual particles, metal leaching, linker migration, degradation products, ecotoxicity, and possible soil accumulation must be carefully evaluated [257]. Therefore, MOFs may be most suitable initially in contained systems, such as fixed-bed adsorbents, immobilized enzyme reactors, or membrane modules, where material retention can be controlled and verified.

MOFs can support circular food systems through enzymatic hydrolysis, inhibitor removal, volatile fatty acid recovery, nutrient capture, catalytic conversion, and closed-loop fermentation [50]. Their greatest potential lies in integrated biorefineries that use selective adsorption, catalytic activity, and reusable biocatalysis to convert real food and fermentation residues into higher-value products.

## 7. Safety, Regulatory Considerations, Scalability, and Future Perspectives

### 7.1. Safety Considerations for MOFs in Food Biotechnology

The translation of MOFs into probiotic delivery, precision fermentation, and circular food systems requires safety assessment that reflects the intended exposure route. Food-related applications differ from biomedical or environmental uses because exposure may be repeated and long-term [101,258]. Therefore, an edible MOF-based probiotic carrier requires stricter evaluation than a MOF immobilized in a closed fermentation column or used as a recoverable adsorbent in wastewater treatment.

MOF safety is influenced by metal-node identity, linker chemistry, particle size, surface charge, degradation behavior, aggregation state, and residual synthesis components [259,260]. For example, ZIF-8 is widely used for biological encapsulation because of its mild synthesis and pH-responsive degradation, but food applications require careful evaluation of zinc release, 2-methylimidazole exposure, and possible effects on intestinal cells or gut microbiota [261,262]. Iron-based frameworks such as MIL-88A may offer stronger food relevance because of their iron-fumarate composition, while highly stable zirconium-based MOFs such as UiO-66 require further evidence regarding long-term dietary exposure and persistence [49,263].

Safety also depends on material format. Free nanoscale MOFs may interact more strongly with biological barriers, whereas MOFs embedded in alginate beads, hydrogels, membranes, or fixed-bed supports may reduce direct exposure if material retention is validated [264]. Thus, the same MOF framework may present different safety implications depending on whether it is ingested, immobilized, or used as a recoverable processing material.

### 7.2. Food-Grade Design and Regulatory Feasibility

Food applications require MOF design to be guided by regulatory plausibility from the beginning. EFSA guidance emphasizes characterization, exposure assessment, hazard identification, toxicokinetics, and uncertainty analysis for nanomaterials in food and feed, while FDA guidance indicates that nanoscale materials may require case-by-case safety evaluation because their properties can differ from conventional forms of the same substance [265]. Therefore, MOFs prepared from familiar metals or organic acids cannot automatically be considered equivalent to their bulk components if the framework exhibits distinct nanoscale behavior.

For edible probiotic delivery, food-grade MOFs should prioritize aqueous synthesis, low toxicity, and nutritionally relevant metal nodes, linkers with established safety profiles, minimal residual solvent, controlled degradation, and clear digestion or elimination pathways. Iron-fumarate frameworks and carbohydrate-based MOFs illustrate more food-compatible design directions, but their stability in gastrointestinal fluids, metal and linker release, epithelial interactions, and microbiota effects still require evaluation [152,266].

Regulatory feasibility will also depend on application format. MOFs directly incorporated into foods or supplements would face stricter requirements than MOFs used as retained processing aids, immobilized fermentation supports, membranes, or packed-bed materials. Thus, near-term translation may be more realistic in contained bioprocessing systems where migration and material carryover can be controlled.

### 7.3. Toxicological and Biological Evaluation Requirements

Robust toxicological evaluation of MOFs for food biotechnology should include both material-centered and biology-centered assays. Material-centered analysis should quantify framework identity, crystallinity, particle-size distribution, zeta potential, morphology, porosity, residual solvents, unreacted metal ions, unbound linkers, degradation products, and metal leaching under relevant pH, ionic strength, and digestion conditions [267]. These parameters are not only characterization details; they define exposure. For example, two ZIF-8 samples with similar X-ray diffraction patterns may differ substantially in particle size, aggregation behavior, residual 2-methylimidazole, and zinc release rate, leading to different biological effects [268].

Biology-centered evaluation should be tailored to the intended application. For edible probiotic carriers, assays should include gastrointestinal digestion models, intestinal epithelial cytotoxicity, oxidative stress, barrier integrity, inflammatory markers, mucus interaction, gut microbiota compatibility, and probiotic recovery after release [269,270]. Caco-2, HT29-MTX, and co-culture intestinal models can provide early screening, but they cannot replace more comprehensive digestion, microbiota, and in vivo studies when direct consumption is proposed.

Nguyen et al. (2025) [271] reported that although ZIF-8-based nanoparticles showed antibacterial potential against *Salmonella Typhimurium* on loose-leaf lettuce, their food application requires careful toxicity assessment because zinc release, 2-methylimidazole exposure, particle persistence, and dose-dependent cytotoxicity may affect consumer safety. For probiotic products, it is also important to evaluate whether MOF encapsulation alters the strain’s beneficial properties, including acid and bile tolerance, adhesion-related traits, antimicrobial metabolite production, and short-chain fatty acid production.

For antimicrobial MOFs, particularly surface-functionalized or metal-doped systems such as Ag- or Zn-containing frameworks, rigorous experimental controls are also necessary to distinguish the intrinsic antimicrobial effects of released metal ions from those associated with the structural framework or surface modification itself. Comparative controls using the parent MOF, equivalent concentrations of free metal ions, and quantitative metal-ion release analysis under identical experimental conditions can help clarify the dominant mechanism of action. Such mechanistic differentiation is essential for accurately interpreting antimicrobial performance, optimizing material design, and supporting the safety and regulatory evaluation of MOF-based antimicrobial technologies for food applications.

For fermentation and circular-system applications, biological evaluation should focus on the process organism, final product, and waste stream. A MOF used to immobilize yeast for precision fermentation should be tested for effects on cell viability, secretion of the target protein, stress responses, mutation pressure, and product purity [132]. On the other hand, a MOF used to recover phosphate from dairy wastewater should be evaluated for regeneration chemicals, metal leaching, and suitability of the recovered nutrient stream for fertilizer or microbial media [272]. Such application-specific evaluation is necessary because a single universal safety test cannot capture the different exposure routes and performance requirements of MOF-enabled food technologies.

### 7.4. Environmental Fate and Life-Cycle Sustainability

MOFs proposed for circular food systems should be evaluated from a life-cycle perspective to ensure that their use does not create new environmental burdens through toxic precursors, solvent-intensive synthesis, poor regeneration, or difficult disposal [271]. For example, MOF-based recovery of volatile fatty acids or phosphate may support resource efficiency, but these benefits could be reduced if the material requires high solvent input, shows metal leaching, or lacks a safe end-of-life pathway.

**Figure 8:**
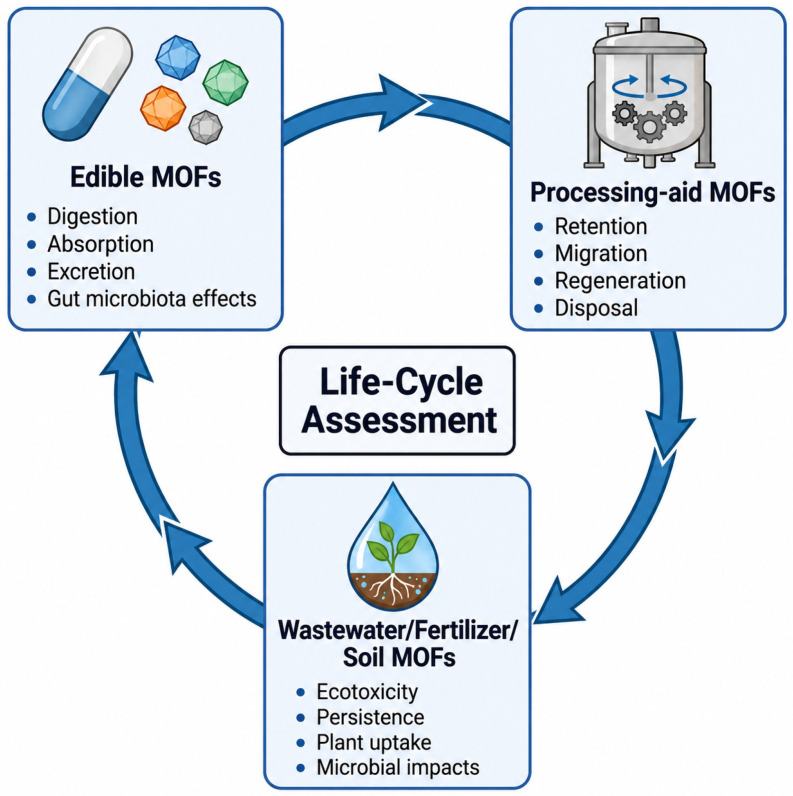
Life cycle sustainability of MOFs in circular food systems.

Environmental fate depends on the application format. Edible MOFs require assessment of digestion, absorption, excretion, and gut microbiota effects, while processing-aid MOFs require evaluation of retention, migration, regeneration, and disposal. MOFs used for wastewater, fertilizer, or soil-related applications should also be assessed for ecotoxicity, persistence, plant uptake, and microbial-community impacts (Figure 8).

Sustainable MOF design should prioritize renewable linkers, benign metals, water-based synthesis, high operational stability, efficient regeneration, and safe degradation. Bio-derived linkers such as fumarate, citrate, amino acids, cyclodextrins, and selected polyphenols may improve alignment with food and circular-economy principles [273,274]. However, sustainability claims should be supported by quantitative data on solvent use, energy demand, yield, regeneration cycles, adsorption performance, material loss, and end-of-life treatment.

### 7.5. Data-Driven Design and Standardization

The broad chemical diversity of MOFs makes conventional trial-and-error development inefficient, particularly for food biotechnology applications where structural, safety, and functional properties must be optimized simultaneously [275]. Data-driven screening and machine learning may accelerate the identification of suitable frameworks for applications such as organic acid recovery, inhibitor removal, enzyme stabilization, and probiotic protection [276].

To support these approaches, future studies should generate standardized, application-relevant datasets that extend beyond structural characterization and model-solution testing. Parameters such as digestion behavior, cytocompatibility, microbial functionality, regeneration, product purity, and residual MOF carryover should be routinely reported. Moreover, MOF-based systems should be benchmarked against established materials, including alginate, chitosan, activated carbon, zeolites, ion-exchange resins, hydrogels, and membranes, to determine whether they provide meaningful advantages in real food and fermentation environments. More broadly, successful translation of MOF-enabled food technologies will require standardized evaluation frameworks that integrate material characterization, biological performance, environmental sustainability, scalability, and long-term safety. Comparable perspectives have recently emerged for catalytic nanozymes used in antimicrobial food systems, emphasizing that harmonized datasets linking material descriptors with application-level performance are essential for artificial intelligence-assisted materials discovery and responsible industrial translation [277]. Similar interdisciplinary recommendations have also been proposed for nanotechnology-enabled environmental remediation, highlighting that the future impact of advanced nanomaterials will depend not only on material innovation but also on rigorous validation and realistic implementation pathways [278].

## 8. Conclusions

This review highlights the growing potential of metal–organic frameworks as multifunctional materials to advance probiotic delivery, precision fermentation, and circular food-system development. Rather than functioning only as porous carriers, MOFs provide tunable interfaces that can be engineered to protect biological components, regulate molecular transport, stabilize enzymes, capture metabolites, and support the recovery of nutrients or value-added compounds from complex food and fermentation streams. These capabilities make MOFs particularly relevant to emerging food biotechnology applications where conventional encapsulation, immobilization, and separation materials may offer limited control over structure, responsiveness, and selectivity.

In probiotic delivery, MOF-based coatings and hybrid nanoarchitectures provide a promising strategy to improve microbial resistance to processing, storage, gastric acidity, bile salts, oxidative stress, and other environmental challenges. Biomimetic mineralization and cytoprotective MOF shells have shown that engineered frameworks can temporarily protect living cells, but their practical use in probiotic foods requires more than improved survival. Future studies should demonstrate strain-specific functionality after release, including metabolic recovery, acid and bile tolerance, adhesion-related properties, antimicrobial activity, and compatibility with real food matrices. MOF–biopolymer systems based on alginate, chitosan, pectin, cellulose, proteins, or hydrogels may provide the most realistic route toward food-compatible probiotic platforms.

In precision fermentation, MOFs may contribute to more efficient and stable bioprocesses through enzyme immobilization, microbial cell stabilization, product inhibition control, metabolite adsorption, and downstream recovery. These functions are particularly valuable for high-value fermentation products such as alternative proteins, enzymes, pigments, flavors, organic acids, and bioactive compounds. However, practical implementation will likely depend on recoverable formats, including enzyme@MOF composites, MOF-hydrogel beads, fixed-bed adsorbents, membranes, and magnetic composites, rather than free nanoparticles dispersed directly into fermentation systems.

MOFs may also support circular food systems by enabling more effective utilization of food-processing residues, fermentation by-products, nutrient-rich effluents, and waste-derived hydrolysates. Relevant opportunities include enzymatic hydrolysis of by-products, removal of inhibitory compounds from hydrolysates, volatile fatty acid recovery, phosphate and ammonium capture, and selective concentration of valuable metabolites. These examples suggest that MOFs could become functional components of integrated food-waste biorefineries when adsorption, catalysis, immobilization, regeneration, and material recovery are considered together.

Despite these opportunities, the field remains at an early stage of translation. Many MOFs reported in the literature were originally developed for biomedical, catalytic, or environmental applications and cannot be directly transferred to food systems without rigorous evaluation. Food-relevant MOFs must be designed using biocompatible metals and linkers, mild and preferably aqueous synthesis routes, low residual solvent content, controlled degradation, and validated recovery or digestion behavior. Key concerns, including metal and linker leaching, cytocompatibility, gut microbiota interactions, sensory effects, environmental fate, scalability, regeneration, and regulatory classification, must be addressed before industrial adoption.

Future progress should be guided by application-specific performance rather than material novelty alone. MOF-based probiotic systems should be benchmarked against established encapsulation materials such as alginate, chitosan, proteins, lipids, and hydrogels. MOF-enabled fermentation systems should be evaluated in real fermentation broths using product titer, yield, productivity, purity, reusability, and material carryover as core metrics. Circular-system applications should be tested using realistic food residues, dairy effluents, fruit and vegetable by-products, spent grain hydrolysates, and precision-fermentation side streams. Through this more rigorous and translational approach, MOFs may evolve from promising laboratory materials into enabling platforms for next-generation functional foods, sustainable fermentation processes, and circular bioeconomy strategies.

## Figures and Tables

**Table 1:** MOF-Based Strategies for In Situ Product Removal and Metabolite Recovery in Fermentation Systems.

Fermentation Challenge	MOF-Based Function	Potential Targets	Key Considerations
Product inhibition reduces cell growth and productivity	In situ adsorption of inhibitory metabolites	Organic acids, alcohols, solvents, fatty acids, aldehydes	High selectivity, reversible adsorption, low toxicity [186]
Accumulation of valuable metabolites in broth	Selective metabolite capture and concentration	Aroma compounds, pigments, phenolics, peptides, enzymes	Tunable pore size, polarity, charge, and coordination sites [187]
Inhibitors from food waste or lignocellulosic hydrolysates	Removal of fermentation inhibitors before or during fermentation	Furans, phenolics, weak acids	Stability in acidic aqueous media and resistance to fouling [188]
Complex broth composition limits adsorbent performance	Composite or immobilized MOF systems	Metabolites in broths containing proteins, salts, polysaccharides, and cells	Anti-fouling design, particle retention, regeneration efficiency [100]
Downstream recovery requires high energy or solvent input	MOF membranes, fixed beds, beads, or columns for separation	High-value food, flavor, enzyme, or fermentation products	Mechanical stability, food-contact safety, low leaching, easy separation [189]

**Table 2:** Potential integration points of MOF applications in circular food systems and food-waste biorefineries.

Process Stage	MOF-Based Role	Example Food-Residue Application	Key Benefit
Pretreatment	Catalytic or enzyme-stabilizing support	Hydrolysis of starch-, cellulose-, or protein-rich residues	Improves substrate accessibility for fermentation [210]
Fermentation	Immobilization matrix or inhibitor-removal material	Supporting enzymes or microbes during the conversion of whey, spent grain, or pomace	Enhances process stability and microbial performance [211]
Inhibitor control	Selective adsorption of toxic by-products	Removal of phenolics, furans, or weak acids from hydrolysates	Improves fermentation efficiency [212]
Product recovery	Adsorbent, membrane, bead, or fixed-bed material	Recovery of volatile fatty acids, organic acids, pigments, phenolics, or amino acids	Concentrate dilute value-added products [213]
Effluent polishing	Nutrient or contaminant capture material	Recovery of phosphate, nitrogen, or residual organics from fermentation wastewater	Supports nutrient recycling and reduces waste discharge [214]

**Table 3:** Applications of MOFs for nutrient recovery from food and fermentation effluents.

Target Nutrient	MOF-Based Recovery Mechanism	Potential Effluent Source	Circular Reuse Pathway
Phosphate	Binding to metal nodes through ligand exchange or inner-sphere complexation	Fermentation wastewater, dairy effluents, digestate streams	Fertilizer formulations or nutrient solutions [31]
Ammonium	Ion exchange, charged sites, or pore-controlled adsorption	Protein-rich food residues, fermentation centrate, anaerobic digestion streams	Nitrogen source for microbial media or fertilizer production [233]
Nitrate	Electrostatic interaction or functionalized linker-based adsorption	Food-processing wastewater or agricultural runoff-linked effluents	Recycled nitrogen source after purification [234]
Organic nitrogen	Selective adsorption or enzyme-assisted fractionation	Spent yeast, peptone-rich broth, microbial biomass residues	Peptides or amino acids for microbial cultivation [235]
Trace minerals	Coordination with metal-binding functional groups	Fermentation broth, food-processing wastewater	Micronutrient recovery for circular agriculture or fermentation media [236]

## Data Availability

No new data was created or analyzed in this study. Data sharing is not applicable to this article.
